# The *Weissella* and *Periweissella* genera: up-to-date taxonomy, ecology, safety, biotechnological, and probiotic potential

**DOI:** 10.3389/fmicb.2023.1289937

**Published:** 2023-12-11

**Authors:** Vincenzina Fusco, Daniele Chieffi, Francesca Fanelli, Marco Montemurro, Carlo Giuseppe Rizzello, Charles M. A. P. Franz

**Affiliations:** ^1^National Research Council, Institute of Sciences of Food Production (CNR-ISPA), Bari, Italy; ^2^Department of Environmental Biology, “Sapienza” University of Rome, Rome, Italy; ^3^Department of Microbiology and Biotechnology, Max Rubner-Institut, Kiel, Germany

**Keywords:** *Weissella*, *Periweissella*, lactic acid bacteria, probiotic, bacteriocin, prebiotic, exopolysaccharides, fermented food

## Abstract

Bacteria belonging to the genera *Weissella* and *Periweissella* are lactic acid bacteria, which emerged in the last decades for their probiotic and biotechnological potential. In 2015, an article reviewing the scientific literature till that date on the taxonomy, ecology, and biotechnological potential of the *Weissella* genus was published. Since then, the number of studies on this genus has increased enormously, several novel species have been discovered, the taxonomy of the genus underwent changes and new insights into the safety, and biotechnological and probiotic potential of weissellas and periweissellas could be gained. Here, we provide an updated overview (from 2015 until today) of the taxonomy, ecology, safety, biotechnological, and probiotic potential of these lactic acid bacteria.

## History and up-to-date taxonomy of *Weissella* and *Periweissella*

The genus *Weissella* was first described by Collins et al. (1993), who isolated a group of *Leuconostoc*-like microorganisms during a survey of the lactic acid microbiota of dry naturally fermented Greek sausage. The novel genus was named *Weissella* M.L. dim. fem. after Norbert Weiss, acknowledged microbiologist which largely contributed to the lactic acid bacteria (LAB) taxonomy. In this first description, seven species were biochemically characterized and differentiated by 16S rRNA gene sequence analysis: *W. confusa* comb. nov. (described in Holzapfel and Kandler, 1969; Kandler and Weiss, 1986) *W. halotolerans* comb. nov. (described in Kandler et al., 1983), *W. kandleri* (described in Holzapfel and van Wyk, 1982), *W. minor* comb. nov. (described in Kandler et al., 1983), *W. paramesenteroides* (described by Garvie, 1967, 1986), *W. viridescens* (described by Niven and Evans, 1957; Kandler and Weiss, 1986), which is the type species of this genus, and *W. hellenica* sp. nov. (Collins et al., 1993). Sources of isolation and type strains are indicated in Table 1.

**Table 1:** List of *Weissella* and *Periweissella* species described to date.

***Species***	**Type strain**	**Source of isolation**	**Reference accession**	**16S rRNA**	**Assigned by**
*P. beninensis*	DSM 22752^T^	Submerged fermenting cassava, Benin	JAGMVS010000000	EU439435	Padonou et al., 2010
*W. bombi*	DSM 28794^T^	Bumble bee gut, Belgium	NZ_FMAO00000000.1	LK054487	Praet et al., 2015
*W. ceti*	CCUG 59653^T^	beaked whales (*Mesoplodon bidens*), USA	NZ_ANCA01000000.1	FN813251	Vela et al., 2011
*W. cibaria*	CCUG 41967^T^	Chili Bo, Malaysia	NZ_VNGZ01000000.1	AJ295989	Björkroth et al., 2002
*W. coleopterorum*	HDW19^T^	intestine of diving beetle (*Cybister lewisianus*), South Korea	NZ_CP049888.1	MN099422	Hyun et al., 2021
*W. confusa*	ATCC 10881^T^	Cabbage, India	NZ_CP027563.1	AB023241	Collins et al., 1993
*P. cryptocerci*	26KH-42^T^	gut of *Cryptocercus kyebangensis*, South Korea	NZ_CP037940.1	MK395366	Heo et al., 2019
*W. diestrammenae*	DSM 27940^T^	gut of a camel cricket (*Diestrammena coreana*), South Korea	JAGMVT010000000	JQ646523	Oh et al., 2013
*P. fabalis*	LMG 26217^T^	Fermented cocoa beans, Brazil	JAGMVU010000000	HE576795	Snauwaert et al., 2013
*P. fabaria*	LMG 24286^T^	Fermented cocoa beans, Ghana	JAGMVV010000000	FM179678	De Bruyne et al., 2010
*P. ghanensis*	DSM 19935^T^	Fermented cocoa beans, Ghana	JAGMVW010000000	AM882997	De Bruyne et al., 2008
*W. fangxianensis*	HBUAS51963^T^	Rice wine starter, China	JAMXDB000000000	OM943160	Xiang et al., 2023
*W. halotolerans*	ATCC 35410^T^	Greek fermented sausages	NZ_ATUU01000000.1	AB022926	Collins et al., 1993
*W. hellenica*	ATCC 51523^T^	Greek fermented sausages	NZ_JAAXPM010000000.1	X95981	Collins et al., 1993
*W. kandleri*	ATCC 51149^T^	Spring in Namib Desert, Namibia	NZ_JQBP01000000.1	AB022922	Collins et al., 1993
*W. koreensis*	CCUG 47134^T^	Kimchi, South Korea	NZ_AKGG01000000.1	AY035891	Lee et al., 2002
*W. minor*	ATCC 35412^T^	Milking machine slime	NZ_JQCD01000000.1	AB022920	Collins et al., 1993
*W. muntiaci*	8 H-2^T^	feces of muntjacs, Formosan barking deer, South Korea	NZ_SDGZ01000000.1	MK774696	Lin et al., 2020
*W. oryzae*	DSM 25784^T^	Fermented rice grain, Japan	NZ_DF820484.1	AB690345	Tohno et al., 2013
*W. paramesenteroides*	ATCC 33313^T^	Human intestinal microbiota	ACKU01000000.1	X95982	Collins et al., 1993
*W. sagaensis*	CCM 8924^T^	Traditional yogurt, China	BLKA01000001.1	LC438526	Li et al., 2020
*W. soli*	CCUG 46608^T^	Garden soil, Sweden	NZ_CP017326.1	AY028260	Magnusson et al., 2002
*W. thailandensis*	CCUG 46557^T^	Fermented fish, Thailand	NZ_BJEC01000000.1	MT760016	Tanasupawat et al., 2000
*W. uvarum*	B18NM42^T^	Grapes, Greek	JAGMVX010000000	KF999666	Nisiotou et al., 2014
*W. viridescens*	ATCC 12706^T^	Cured meat product, China	NZ_CP061835.1	X52568	Collins et al., 1993

In 2000, Tanasupawat et al. (2000) isolated some LAB from fermented fish in Thailand whose DNA–DNA genetic relatedness toward the previously described *Weissella* species allowed the authors to assign them as *W. thailandensis* sp. nov. and identified the type strain as FS61-1^T^. In 2002, three additional species were described and included into the *Weissella* genus: *W. cibaria, W. soli*, and *W. koreensis*. *W. cibaria* was described by Björkroth et al. (2002): The authors selected 37 isolates, from humans and animal clinical samples as well as from foods sold in Malaysia and differentiated this species based on DNA–DNA reassociation experiments, which showed hybridization levels below 49% toward *W. confusa*. The type strain of this species is *W. cibaria* CCUG 41967^T^, which was isolated from the popular food ingredient Chili Bo. *W. soli* was isolated from garden soil by Magnusson et al. (2002) and showed relatedness to *W. kandleri* and *W. confusa* (95.5 and 95.3% 16S rRNA gene sequence identity, respectively). Lee et al. (2002) isolated from kimchi (a Korean fermented vegetable food) some strains with 97.2% 16S rRNA gene sequence identity to *W. kandleri*. The novel species was named *W. koreensis*, and the designated type strain was KCTC 3621^T^.

In 2010, Padonou et al. (2010) characterized the novel species *W. beninensis* sp. nov., isolated from submerged fermenting cassava in Ketou, Benin, and, since this species was demonstrated as being motile, they emended the description of *Weissella* genus that until then only comprised non-motile species. *W. fabaria* and *W. fabalis* were described in De Bruyne et al. (2010) and Snauwaert et al. (2013), respectively. *W. fabaria* LMG 24289^T^ was isolated from traditional heap fermentations of Ghanaian cocoa beans and, although it showed 99.5% 16S rRNA gene sequence identity toward *W. ghanensis* LMG 24286^T^, DNA–DNA hybridization and metabolic characteristics recognize them as a separate species. *W. fabalis* LMG 26217^T^ was isolated from a Brazilian cocoa bean fermentation and had the highest 16S rRNA gene sequence identity toward *W. fabaria* LMG 24289^T^ (97.7 %). The confirmation that this strain could have been recognized as a novel species was achieved by *phe*S gene sequence analysis, DNA–DNA hybridization, the MALDI-TOF MS profile, and biochemical analysis.

*W. diestrammenae* was isolated from the gut of a camel cricket (*Diestrammena coreana*) in 2013 by Oh et al. (2013) and showed the highest 16S rRNA gene sequence identity to *W. koreensis* (97.7 %). The type strain of this species is *W. diestrammenae* DSM 27940^T^. In the same period, Tohno et al. (2013) conducted a taxonomic study on SG25^T^ and SG23. These LAB strains, Gram-positive and catalase-negative, were isolated from grains of fermented Japanese rice (*Oryza sativa* L. subsp. *japonica*). Genetic analysis showed that *W. soli* was the closest relative to this novel *W. oryzae* species (96.9% of 16S rRNA gene sequence identity). *W. uvarum* was described in 2014 by Nisiotou et al., who isolated this species while studying the microbiota of wine grapes (*Vitis vinifera* L.) from the Nemea region in Greece. Although *W. uvarum* 16S rRNA gene sequence showed a high identity to that of *W. minor* NRIC 1625^T^ (99.1 %), the isolated strains were assigned to a novel species for their capacity to grow at 42°C and to ferment specific carbohydrates, by using a genetic approach including randomly amplified polymorphic DNA (RAPD), restriction enzyme analysis-pulse field gel electrophoresis (REA-PFGE), and repetitive sequence-based PCR (rep-PCR analyses).

The name *W. bombi* was assigned by Praet et al. (2015) to a novel LAB isolated from the bumble bee gut in the region of Ghent, Belgium, which showed a 99.6% 16S sequence identity to that of *W. hellenica* LMG 15125^T^. The analysis of the *phe*S gene sequences, DNA G+C content analysis, (GTG)_5_-PCR fingerprinting, DNA–DNA hybridization experiments, and a biochemical characterization led to the assignment of this novel species, whose type strain is LMG 28290^T^. *W. ceti* was first isolated in 2011 by Vela et al. (2011) within a study aimed at assessing the microbiota of beaked whales (*Mesoplodon bidens*). The type strain of this species is CCUG 59653^T^. In 2019, Heo et al. (2019) isolated from the gut of an insect, *Cryptocercus kyebangensis* sampled from mountains in South Korea, a novel organism that in a 16S rRNA gene phylogenetic tree analysis clustered with *W. ghanensis, W. beninensis, W. fabaria*, and *W. fabalis*-type strains and showed a sequence identity of 95.9% with both *W. beninensis* 2L24P13^T^ and *W. ghanensis* LMG 24286^T^. Genomic and phenotypic analyses allowed the description of the novel species *W. cryptocerci*.

*W. muntiaci* was characterized by Lin et al. (2020), who isolated this Gram-positive strain from feces of a barking deer (*Muntiacus reevesi*) in Taiwan. *W. muntiaci* 8H-2^T^ showed a 16S rRNA gene sequence identity with the type strains of *W. oryzae, W. confusa, W. cibaria*, and *W. soli* of 99.2, 97.8, 97.6, and 97.3 %, respectively. In the same year, Li et al. (2020) characterized three Gram-positive bacterial strains, named as X0750^T^, X0401, and X0278. The type strain, isolated from a traditional yogurt of the Chines Saga County, showed a 16S rRNA gene sequence 94.4–100 % identical to that of the type strains of *W. hellenica, W. bombi, W. paramesenteroides, W. jogaejeotgali, W. thailandensis, W. oryzae, W. cibaria*, and *W. confusa*. The resulting novel species was designated as *W. sagaensis*.

In 2021, Hyun et al. (2021) isolated some weissellas from the intestine of the diving beetle *Cybister lewisianus* that showed 98.9% 16S rRNA gene sequence identity and 79.5% ANI to *W. koreensis* KCTC 3621^T^. Based on phylogenetic, chemotaxonomic, phenotypic, and genotypic analyses, named this novel species *W. coleopterorum*, whose type strain is HDW19^T^.

Until 2022, the genus counted 26 species, including *W. jogaejeotgali* which was described by Lee et al. (2015b), but then was identified as a later heterotypic synonym of *W. thailandensis*, described by Tanasupawat et al. (2000), as well as *W. kimchi*, which was first described by Choi et al. (2002), but later correctly identified as the later heterotypic synonym of *W. cibaria* (Ennahar and Cai, 2004), described by Björkroth et al. (2002).

In 2022, Bello et al. (2022) revised the taxonomy of the genera *Leuconostoc, Convivina, Oenococcus, Fructobacillus*, and *Weissella* by using the concatenated sequences of 498 core proteins and the 16S rRNA gene phylogeny. The authors then transferred five species that clustered in a separate clade from the genus *Weissella* into the proposed novel genus *Periweissella* (*P*.): *P. cryptocerci* (basonym *W. cryptocerci*; Heo et al., 2019), *P. beninensis* (basonym *W. beninensis*; Padonou et al., 2010), *P. fabalis* (basonym *W. fabali*s; Snauwaert et al., 2013), *P. fabaria* (basonym *W. fabaria*; De Bruyne et al., 2010), and *P. ghanensis* (basonym *W. ghanensis*; De Bruyne et al., 2010) (Table 1). The same assumption was achieved by Fanelli et al. (2022) who, in the same year, showed that *Weissella* species could be clustered into six different species groups by a genome-based phylogenomic analysis, with one including the five species that Bello proposed as belonging to a separate novel genus. Furthermore, the phylogenomic species group clustering, in many cases, overlapped with the carbohydrate metabolism pathways (Fanelli et al., 2022).

In 2023, a novel species, namely *W. fangxianensis* sp. nov., was described by Xiang et al. (2023), who isolated three LAB strains from rice wine starter used in Fangxian County (China). This novel species was described using a polyphasic approach, based on genomic and metabolic analyses. Both 16S rRNA gene sequence and genomic phylogeny placed this species close to *W. thailandensis* and *W. paramesenteroides*. The type strain of this species is HBUAS51963^T^ (Table 1).

Both the *Weissella* and *Periweissella* genera are members of the phylum *Bacillota*, class *Bacilli*, order *Lactobacillales*, and family *Lactobacillaceae*, recently revised by Zheng et al. (2020), who merged it with the *Leuconostocaceae* family.

## Descriptions of species in the genus *Weissella*

All *Weissella*, such as other LAB, are Gram-positive and catalase-negative. They occur in short rods with tapered rounded ends or ovoid cells (Collins et al., 1993; Björkroth et al., 2014), and they have a tendency toward pleomorphism. Weissellas do not produce spores, and they are facultatively anaerobic chemoorganotrophs fermenting glucose heterofermentatively by way of the hexose-monophosphate and phosphoketolase pathways that deliver lactic acid as end products [dl or d(-), depending on the species], carbon dioxide, and ethanol and/or acetic acid. For their growth, weissellas have an obligate need for vitamins, peptides, amino acids, fatty acids, nucleic acids, and fermentable carbohydrates. Not all *Weissella* species hydrolyze arginine. Growth at 15°C has been detected for all weissellas, while only some species are able to grow at 42–45°C (Björkroth et al., 2014). All *Weissella* strains are able to utilize maltotriose, d-fructose, N-acetyl-d-glucosamine, turanose, d-mannose, and α-d glucose palatinose. The peptidoglycan of weissellas typically contains lysine as diamino acid and apart from *W. kandleri* which contains also glycine, and all weissellas contain alanine or alanine and serine in the interpeptide bridge (Holzapfel and van Wyk, 1982).

There are currently 20 *Weissella* species which, based on phylogenomic comparison by Fanelli et al. (2022) and the genomic data available for the *W. fangxianensis* species (Xiang et al., 2023), can be grouped into five species groups, i.e., the *W. kandleri* species group (*W. kandleri, W. soli, W. diestrammenae, W. coleopterorum, W. koreensis*), the *W. oryzae* species group (*W. oryzae, W. muntiaci*), the *W. halotolerans* group (*W. halotolerans, W. ceti, W. uvarum, W. minor, W. viridescens*), the *W. confusa* species group (*W. confusa, W. cibaria*), and the *W. paramesenteroides* species group (*W. thailandensis, W. fangxianensis, W. paramesenteroides, W. bombi, W. hellenica, and W. sagaensis*). The description of the type species and species in alphabetical order follows below.

Description of the type species:

## *Weissella viridescens* (Niven and Evans, 1957) Collins, Samelis, Metaxopoulos, and Wallbanks 1993 601^AL^

Synonyms: *Lactobacillus viridescens* Niven and Evans 1957, p. 758; *Lactobacillus corynoides* subsp. *corynoides* Kandler and Abo-Elnaga, 1966, p. 573. Note that in the Approved List of Bacterial Names *L. viridescens* is incorrectly cited *as Lactobacillus viridescens* Kandler and Abo-Elnaga 1966, p. 573. For this strain, the utilization of sucrose concomitant with a possible production of dextran has not been studied.

vi.ri.des'cens. M.L. pres. part. *viridescens*, growing green, greening.

*W. viridescens* cells, which are non-motile, appear as irregular rods, occurring singly or in pairs, with rounded tapered ends. The interpeptide bridge of the peptidoglycan contains lysine, alanine, and serine. *W. viridescens* produces dl lactic acid from glucose, whereas it does not utilize arginine. The type strain (ATCC 12706^T^ = DSM 20410^T^ = LMG 3507^T^) was isolated from a discolored meat product. The mol% GC of the DNA is 41–44, and the GenBank accession nos. for the 16S rRNA gene sequence are ABO23236, M23040, and X52568.

Description of other *Weissella* species:

## *Weissella bombi* Praet, Meeus, Cnockaert, Houf, Smagghe, and Vandamme 2015, 2022^VP^

bom'bi. L. n. *bombus* a boom, a deep hollow noise, buzzing, also the zoological genus name of the bumble bee: N.L. gen. n. *bombi* of *Bombus*, of a bumble bee.

Cells, which are non-motile, occur as elongated cocci, in pairs or chains. *W. bombi* does not grow at 45°C and produces d(-) lactic acid from glucose. The type strain, isolated from the gut of a *B. terrestris* bumble bee, is LMG 28290^T^ (=DSM 28794T). The mol% GC content of the DNA is 37.2, and the GenBank 16S rRNA gene sequence accession number is LK054487.

## *Weissella ceti* Vela, Fernández, Bernaldo de Quirós, Háerrez, Domínguez, and Fernández-Garayzábal, 2011, 2760^VP^

ce.ti. L. gen. n. *ceti* of a whale.

*W. ceti* are short rod-shaped or coccoid cells, which are non-motile and occur singly or in pairs. It can grow at 22°C and 37°C but not at 15°C or 42°C. *W. ceti* produces dl lactic acid from glucose and does not produce dextran from sucrose. The type strain hydrolyzes arginine, while this feature is variable among the other strains of this species. The type strain, isolated from the spleen of a beaked whale (*Mesoplodon bidens*), is CECT 7719^T^ = LMG 30639^T^. The DNA G+C content of the type strain is 39.2 mol%, and the GenBank accession no. of the 16S rRNA nucleotide sequence is FN813251.

## *Weissella cibaria* Björkroth, Schillinger, Geisen, Weiss, Hoste, Holzapfel, Korkeala, and Vandamme 2002, 147^VP^

ci.ba'ri.a. L. adj. *cibaria*, pertaining to food.

*W. cibaria* cells are non-motile and occur as short rods in pairs. The interpeptide bridge structure is Lys-Ala-(Ser)-Ala. *W. cibaria* produces dl lactic acid from glucose and grows at 45°C and 15°C but not at 4°C. It produces ammonia from arginine and dextran from sucrose. The type strain, isolated from Malaysian chili bo, is DSM 15878^T^ = LMG 17699^T^. It has a mol% GC content of the DNA of 44, and the 16S rRNA gene sequence GenBank accession n. is AJ295989.

## *Weissella coleopterorum* Hyun, Lee, Sung, Kim, Jeong, Lee, Yun, Choi, Han, Lee, Tak, Kim, and Bae 2021, 10^VP^

co.le.o.pte.ro'rum. N.L. gen. pl. n. *coleopterorum* of Coleoptera, the name of the order of the beetles from which the strain was first isolated.

*W. coleopterorum* cells are typically non-motile and rod-shaped. It grows at 4–37°C. The amino acids in the peptidoglycan structure are alanine and lysine. Its DNA has a mol% GC content of 37.2%. The type strain isolated from the intestine of the diving beetle *Cybister lewisianus* is JCM 33684^T^ = KCTC 43114^T^. The GenBank/EMBL/DDBJ accession number of the 16S rRNA gene sequence of the type strain is MN099422.

## *Weissella confusa* Collins, Samelis, Metaxopoulos, and Wallbanks 1993, 599^AL^

Synonyms: *Lactobacillus confusus* Garvie and Tilbury 1972, p. 396; *Lactobacillus coprophilus* subsp. *confusus* Holzapfel and Kandler 1969, p. 665.

con.fu'sus. L. v. *confundere* to confuse: L. past. part. *confusus* confused.

*W. confusa* has non-motile cells occurring as short rods, singly or (rarely) in short chains, with a tendency to be thicker at one of the ends. Lys-Ala is the amino acid that occurs in the interpeptide bridge of the peptidoglycan structure. The lactic acid enantiomers produced from glucose metabolism are dl. Growth at 45°C is variable with some strains capable of good growth at this temperature. Dextran is produced from sucrose and ammonia from arginine. The type strain, isolated from sugar cane, is DSM 20196^T^ = ATCC 10881^T^ = LMG 9497^T^. The DNA has a mol% GC content of 45–47, and the 16S rRNA gene sequence GenBank accession nos. are AB023241 and M23036.

## *Weissella diestrammenae* Oh, Shin, Hyun, Kim, Kim, Kim, Yun, and Bae 2013, 2954^VP^

di.es.tram.me'nae. N.L. gen. n. *diestrammenae* of *Diestrammina*, referring to *Diestrammena coreana*, a camel cricket from the gut of which this bacterium was isolated.

*W. diestrammenae* does not exhibit motility, and the cell's morphologies are coccoid or rod-shaped. It is capable of growth at 4–37°C in 0 to 4% NaCl. The bacteria can hydrolyze arginine to ammonia and produce d(-) lactic acid from glucose. The amino acids in the cell wall are Lys-Ala-Ser. The DNA has mol% GC content of 45, and the type strain, isolated from the gut of a camel cricket (*Diestrammenae coreana*) in South Korea, is JCM 18559^T^ = DSM 27940^T^ = LMG 30643^T^. The GenBank/EMBL/DDBJ accession number for the 16S rRNA gene of the type strain is JQ646523.

## *Weissella fangxianensis* Xiang, Dong, Cai, Zhao, Liu, Shan, and Guo 2023, e005870, 7^VP^

fang. xian. en'is. N.L. fem. adj. fangxianensis pertaining to Fangxian county, a city located in Hubei Province, PR China, where the type strain was first isolated.

Cells are non-motile, non-spore-forming and spherical. Growth occurs at 15–37°C but not at 10°C or 40°C, with optimum between 25 and 35°C. The genomic DNA mol% GC content is 38.6 mol%. The type strain, isolated from rice wine starter in Fangxian county Hubei Province, PR China, in 2021, is GDMCC 1.3506^T^ = JCM 35803^T^.

## *Weissella halotolerans* Collins, Samelis, Metaxopoulos, and Wallbanks 1993, 599^VP^

Synonym: *Lactobacillus halotolerans* Kandler, Schillinger and Weiss, 1983, 672. Effective publication: kandler, Schillinger and Weisss, 1983, p. 283.

ha.lo.to'le.rans. Gr. n. *hals, halos* salt; L. part. adj. *tolerans*, tolerating, enduring; N.L. part.adj. *halotolerans*, salt-tolerating.

*W. halotolerans* does not exhibit motility and cells appear as irregular, short or even coccoid rods, with rounded to tapered ends with a tendency to form coiling chains. Cells were observed also to clump together. Lys-Ala-Ser is the amino acid composition of the interpeptide bridge of the peptidoglycan structure. *W. halotolerans* produces dl lactic acid from glucose and does not grow at 45°C, whereas it grows in 12% NaCl, with very weak growth occurring at 14% NaCl. Arginine is not metabolized, and dextran production from sucrose has not been investigated.

## *Weissella hellenica* Collins, Samelis, Metaxopoulos, and Wallbanks 1993

hel.le'ni.ca. Gr. masc. adj. *hellênikos*, Greek; N.L. fem. adj. *hellenica*, Greece, from where the bacterium was first isolated.

The non-motile cells of this species are spherical but sometimes also show a lenticular morphology and generally occur in pairs or short chains. A tendency to associate in clusters was observed. *W. hellenica* grow at 10°C and 4°C (delayed) but not at 37°C. All strains produce d(-) lactic acid from glucose. *W. hellenica* does not hydrolyze arginine and does not produce slime from sucrose. The cell wall murein is type Lys-L-Ala-L-Ser(L-Ala). The DNA base compositions of strains ranged between 39.4 and 40.0 mol% GC, respectively, and the type strain, isolated from fermented sausages, is NCFB 2973^F^ = DSM 7378^T^ = LMG 15125^T^. The 16S ribosomal gene GenBank accession number is NR_118771.1.

## *Weissella kandleri* Collins, Samelis, Metaxopoulos, and Wallbanks 1993, 599^VP^

Synonym: *Lactobacillus kandleri* Holzapfel and van Wyk 1983, 439. Effective publication: Holzapfel and van Wyk 1982, 501.

kand'le.ri. M.L. gen. n. *kandleri*, of Kandler; named for O. Kandler, a German microbiologist.

*W. kandleri* cells were observed to be non-motile of partly irregular rod shape, and they were found to occur singly as well as in in pairs but seldom in short chains. Lys-Ala-Gly-Ala_2_ was the amino acid determined to occur in the interpeptide bridge of the peptidoglycan structure. *W. kandleri* produces dl lactic acid from glucose, ammonia from arginine, and dextran from sucrose. It does not grow at 45°C. The type strain, isolated from a desert spring, is DSM 20595^T^ = LMG 18979^T^ which has a 39 mol% GC content in the DNA. The GenBank accession numbers of the 16S rRNA gene are AB022922 and M23038.

## *Weissella koreensis* Lee, Lee, Ahn, Mheen, Pyun, and Park 2002, 1260^VP^

ko.re.en'sis. N.L. adj. *koreensis* of Korea, where the novel organisms were isolated.

Cells are irregular, short, and rod-shaped or coccoid. No growth occurs at 42°C, but it grows at 10 and 37°C. It is capable of arginine hydrolysis and production of dextran from sucrose and d(-) lactic acid from glucose metabolism. The DNA has a G-C content of 37 mol%, and the cell wall was shown to contain Lys-Ala-Ser. The type strain, isolated from the traditional Korean fermented vegetable kimchee, is DSM 15830^T^ = KCCM 41516^T^ = JCM 11263^T^. The 16S rDNA sequences of the type strain have the GenBank/EMBL/DDBJ accession number AY035891.

## *Weissella minor* Collins, Samelis, Metaxopoloulos, and Wallbanks 1993, 599^VP^

Synonyms: *Lactobacillus minor* (Kandler, Schillinger and Weiss 1983, 672. Effective publication: Kandler, Schillinger and Weiss, 1983, 284. (*Lactobacillus corynoides* subsp. minor Abo-Elnaga and Kandler 1965, 128; *Lactobacillus viridescens* subsp. minor Kandler and Abo-Elnaga, 1966, 754).

mi'nor. L. comp. adj. *minor* smaller.

*W. minor* shows non-motile cells appearing as irregular short rods, occurring in pairs or short chains, with rounded to tapered ends often bent with unilateral swellings. The amino acid composition of the peptidoglycan structure is Lys-Ser-Ala_2_. It produces dl lactate from glucose and ammonia from arginine but does not produce dextran from sucrose. It does not grow at 45°C. The mol% GC content of the DNA is 44, and the type strain, which stems from the sludge of milking machines, is DSM 20014^T^ = LMG 9847^T^. The GenBank accession no. of the 16S rRNA gene is M23039.

## *Weissella muntiaci* Lin, Wang, Wu, Guu, Tamura, Mori, Huang, and Watanabe 2020, 1581^VP^

mun.ti′a.ci. N.L. gen. n. *muntiaci* of *Muntiacus*, a genus of the muntjacs, barking deer of Taiwan, from which the type strain was isolated.

*W. muntiaci* shows non-motile cells appearing as short rods occurring singly and rarely in pairs. It grows at 10 to 37°C but not at 4, 45, and 50°C. It produces ammonia from arginine and d(-) lactic acid from glucose. The amino acid composition of the peptidoglycan structure is A3α (l-Lys–l-Ala–l-Ser) with the presence of Glu, Ser, Ala, and Lys in a molar ratio of 1: 1: 3:1. The type strain was obtained from the feces of the Formosan barking deer. This feces were collected in the Fushan Botanical Garden, Yilan County, Taiwan, in 2017. The type strain is BCRC 81133^T^ = NBRC 113537^T^. The genomic mol% GC content is 40.5. The 16S rRNA gene sequence of the type strain has the GenBank/EMBL/DDBJ accession number MK774696.

## *Weissella oryzae* Thono, Kitahara, Inoue, Uegaki, Irisawa, Ohkuma, and Tajima 2013, 1418^VP^

o.ry'za.e. L. gen. n. *oryzae* of rice, from which the type strain was isolated.

*W. oryzae* shows non-motile cells that are irregular, short rod-shaped, or coccoid and occur singly or in pairs and/or short chains. The peptidoglycan structure consists of glutamic acid, lysine, serine, and alanine. *W. oryzae* grows at 10–42°C but not at 4 or 50°C. It is a facultatively anaerobic lactic acid bacterium that does not produce dextran from sucrose and produces d(-) from glucose. The type strain DSM 25784^T^ = LMG 30913^T^ originates from fermented rice grain that was obtained in Tochigi, Japan. This strain has a DNA with mol%GC content of 40.6 mol%, while its 16S rRNA gene sequence has the GenBank/EMBL/DDBJ accession number AB690345.

## *Weissella paramesenteroides* Collins, Samelis, Metaxopoulos, and Wallbanks 1993, 601^AL^ (*Leuconostoc paramesenteroides* Garvie 1967, p. 446)

*pa.ra.me.sen.ter.oi'des*. Gr. prep. *para* resembling; M.L. *mesenteroides* a specific epithet; M.L. adj. *paramesenteroides*, resembling *Leuconostoc mesenteroides*.

*W. paramesenteroides* has non-motile cells that are spherical but often also lenticular, occurring in pairs and chains. It grows at 30°C but optimally at 18–24°C. Lys-Ala_2_ or Lys-Ser-Ala_2_ is the amino acid occurring in the peptidoglycan structure. *W. paramesenteroides* produces d(-) lactate from glucose but not ammonium from arginine and dextran from sucrose. The DNA of the species has a mol% GC content ranging from 37 to 38, and the type strain, that originated from a dairy source, is DSM 20288^T^ = LMG 9852^T.^ The GenBank accession nos. for the 16S rRNA gene are AB023238, M23033, and X95982.

## *Weissella sagaensis* Li, Tian, and Gu 2020, 2491^VP^

sa. ga. en′sis. N.L. fem. adj. *sagaensis*, pertaining to Saga County, a county located in Tibet Autonomous Region, PR China, where the bacterium was isolated.

*W. sagaensis* are non-motile cells which appear as rods that can occur singly, in pairs or in short chains. This bacterium can grow at 10–37°C but not at 5 or 45°C. It produces d(-) lactate from glucose and does not hydrolyze arginine. The interpeptide bridge contains serine and alanine. The DNA of the type strain has a mol% GC of 36.7. The culture collection numbers for the type strain are NCIMB 15192^T^ = LMG 31184^T^, and the 16S rRNA gene sequence is stored in GenBank/ENA/DDBJ under the accession number LC438526.

## *Weissella soli* Magnusson, Jonsson, Schnürer, and Roos 2002, 833^VP^

so'li. L. n. *solum* soil; L. gen. n. *soli*, of the soil.

*W. soli* shows non-motile cells, occurring singly or in pairs, that are rod-shaped and often thickened at one end. The composition of the interpeptide bridge of the peptidoglycan structure is not known. *W. soli* produces d(-) lactate from glucose, dextran from sucrose, and not ammonia from arginine. It grows at 4–40°C but not at 45°C. The type strain that stems from soil is DSM 14420^T^ = LMG 20113^T^, and the DNA of this strain has a mol% GC content of 43. The GenBank accession no. of the 16S rRNA gene is AY028260.

## *Weissella thailandensis* Tanasupawat, Shida, Okada, and Komagata 2000, 1484^VP^

thai.lan'den.sis M.L. fem. adj. *thailandensis* pertaining to Thailand, where the strains were first isolated.

*W. thailandensis* occurs as non-motile coccoid cells arranged either in pairs or chains. d(-) is the major lactic acid enantiomer produced from glucose. It does not hydrolyze arginine and does not produce slime from sucrose. It grows at 25 to 37°C but not at 42°C, and the peptidoglycan structure contains l-Lys-l-Ala. The strains of this species have mol% GC contents of their DNA ranging from 38 ± 0 to 41.2. The type strain stems from pla-ra which is a Thai fermented fish and received the culture collection numbers DSM 15832^T^ = LMG 19821^T^ = JCM 10695^T^. The DDBJ accession number for the 16S rRNA gene sequence of the type strain is AB023838.

## *Weissella uvarum* Nisiotou, Dourou, Filippoussi, Banilas, and Tassou 2014, 3889^VP^

u.va'rum. L. fem. gen. pl. n. *uvarum* of grapes, where the type strain was isolated.

*W. uvarum* has non-motile cells appearing as cocci or short rods that occur singly, in pairs or short chains. It can grow at both 15 and 42°C but not at 4 or 45°C. It produces d(-) lactate from glucose, ammonia from arginine, and not dextran from sucrose. The type strain of this species is the only strain among weissellas and periweissellas to utilize d-arabitol and d-sorbitol (Fanelli et al., 2022). The mol% GC of the DNA is 39.1, and the type strain, isolated from grapes from the region of Nemea located in Greece, is DSM 28060^T^ = LMG 30647^T^. The GenBank/EMBL/DDBJ accession number for the 16S rRNA gene sequence of the type strain is KF999666.

## Description of the genus *Periweissella*

Bello et al. (2022) showed *Weissella* species to occur in two distinct clades in a core protein tree derived from the genomes, and comparative analyses, furthermore, identified various conserved signature indels in signature specific for the members of the two clades. The Weissellas, therefore, could be shown not to constitute a monophyletic group but instead comprise two distinct and unrelated clades, namely, a “main clade” and “clade 2.” The clade 2 *Weissellas* shared the presence of five conserved signature indels in the proteins amidophosphoribosyltransferase protein, DEAD/DEAH box helicase, ArgR family transcriptional regulator, Flp pilus assembly complex ATPase component (TadA), and hydroxyethylthiazole kinase. Species of clade 2 were thus proposed to belong to a novel genus, i.e., *Periweissella* gen. nov. (Bello et al., 2022). Periweissellas are Gram-positive and obligately heterofermentative bacteria that appear as non-spore-forming short rods or cocci. They grow at temperatures 15–37°C (optimum 28–30°C), and their DNA has a mol% GC content ranging from 35.4 to 41.1. Several species of this genus may hydrolyze arginine (Bello et al., 2022). The *P. beninensis, P. fabalis-, P. fabaria-*, and *P. ghanensis*-type strains are capable of utilizing α-ketobutyric acid, glycyl-l-methionine, and pyruvic acids, while only *P. fabaria* and *P. fabalis* are able to utilize d-malic acid, i-erythritol, and d-trehalose. The *P. fabaria*-type strain is the only strain among these which is able to utilize formic acid, while the *P. fabalis*-type strain metabolizes fumaric acid, glycyl-l-glutamine, and α-cyclodextrin (Fanelli et al., 2022).

Recent studies demonstrate that with the exception of *P. cryptocerc*i, all *Periweissella* species possess genetic loci coding for flagellar-related proteins (Fanelli et al., 2023a; Qiao et al., 2023), and flagellar structures have been detected in *P. beninensis-, P. ghanensis-, P. fabalis-*, and *P. fabaria*-type strains (Qiao et al., 2023).

Pe. ri. weiss. el'la. Gr. prep. *peri*, about, around or nearby; N.L. fem. dim. n. *Weissella*, a bacterial genus named after Norbert Weiss, a German microbiologist; N.L. fem. dim. n. *Periweissella*, a genus about or nearby *Weissella*.

Description of the type species:

## *Periweissella ghanensis* Bello, Rudra, and Gupta 2022, 16^VP^

Synonym *Weissella ghanensis* De Bruyne, Camu, Lefebvre, de Vuyst, and Vandamme 2008, 2723^vp^

gha.nen'sis. N.L. fem. adj. *ghanensis*, pertaining to Ghana.

Cells are small rods appearing singly, in pairs or short chains. The type strain produces both the dl lactic acid enantiomers (dl 90:10) from glucose. *W. ghanensis* produces ammonium from arginine and slime from glucose. The mol% GC content of the DNA is 40.0, and the type strain (DSM 19935^T^ = LMG 24286^T^) was isolated from Ghanaian cocoa heaps undergoing fermentation. The GenBank accession no. of the 16S rRNA gene is AM882997.

Description of other species:

## *Periweissella beninensis* Bello, Rudra, and Gupta 2022, 16^VP^

Synonym *Weissella beninensis* Padonou, Schillinger, Nielsen, Franz, Hansen, Hounhouigan, Nago, and Jakobsen, 2010, 2196^VP^

ben.in.en'sis. N.L. fem. adj. *beninensis*, pertaining to Benin.

*P. beninensis* exhibits motility, and the cells were shown to possess with peritrichous flagella. Cells were determined to be short and rod-shaped or coccoid. Cells were observed to occur singly, in pairs or short chains. The bacterium is capable of growth at 15°C but not at 45°C. It hydrolyzes arginine and produces dl lactate from glucose. Most strains were observed to produce dextran from sucrose. Among weissellas and periweissellas, *P. beninensis* utilizes the widest range of carbohydrates tested (Fanelli et al., 2022). Indeed, the type strain of this species metabolizes α-d-lactose, d-melibiose, d-galactose, β-methyl-d-galactoside, pyruvic acid methyl ester, lactulose, sucrose, uridine-5'-monophosphate, and d-raffinose (Fanelli et al., 2022). The type strain was isolated from cassava fermentations in Ketou, Benin, and the mol% GC content is 37. The type strain is DSM 22752^T^ (=LMG 25373^T^). The GenBank accession no. for the 16S rRNA gene sequence is EU439435.

## *Periweissella cryptocerci* Bello, Rudra, and Gupta 2022, 16^VP^

Synonym *Weissella cryptocerci* Heo, Hamada, Cho, Weon, Kim, Hong, Kim, and Kwon 2019, 2805^VP^

cryp.to.cer'ci. N.L. gen. n. *cryptocerci*, of *Cryptocercus*, a genus of insect from which the species was isolated.

*P. cryptocerci* dos not exhibit motility, and cells appear to be rod-shaped. The bacterium grows at 4–35°C and can produce dl lactate from glucose. It does not hydrolyze arginine and does not produce slime from sucrose. The cell wall peptidoglycan is type A4α, characterized by an interpeptide bridge of Gly-D-Glu. The mol% GC content is 41.1, and the type strain, isolated from the gut of the insect *Cryptocercus kyebangensis*, was obtained in the mountainous area of Seoraksan, Yangyang-gun, Republic of Korea. The type strain was deposited as KACC 18423^T^ = NBRC 113066^T^. Its GenBank accession number of the 16S rRNA gene is MK395366.

## *Periweissella fabalis* Bello, Rudra, and Gupta 2022, 17^VP^

Synonym *Weissella fabalis* Snauwaert, Papalexandratou, De Vuyst, and Vandamme 2013, 1714^VP^

fa.ba'lis. L. fem. adj. *fabalis* of or belonging to beans.

*P. fabalis* did not show motility, and the cells were observed to be of coccoid morphology, occurring singly, in pairs or in short chains. The bacterium can grow at temperatures ranging from 15 to 37°C and in the presence of 5–6% NaCl but not in the presence of 7–8% NaCl. It produces ammonia from arginine and d-lactic acid from glucose. The DNA of the type strain has a mol% GC content of 37. The type strain, isolated from a Brazilian cocoa bean box fermentation carried out in Ilhéus, Bahia, Brazil, in 2007, is LMG 26217^T^ = DSM 28407^T^. The 16S rRNA gene nucleotide sequence has the GenBank/EMBL/DDBJ accession number HE576795.

## *Periweissella fabaria* Bello, Rudra, and Gupta 2022, 17^VP^

Synonym *Weissella fabaria* De Bruyne, Camu, De Vuyst, and Vandamme 2010, 2002^VP^

fa.ba'ri.a. L. fem. adj. *fabaria* of or belonging to beans.

*P. fabaria* was described to be non-motile, and the cells were determined to have a coccoid morphology, occurring singly, in pairs or short chains. Bacteria of this species produce both the d and l lactic acid enantiomers in a ratio 9:1. They grow at 15–37°C and produce ammonia from arginine and slime from glucose. L-Lys–L-Ala–L-Ser is the amino acid present in the peptidoglycan structure. The DNA of the type strain has a mol% GC content of 38.2 mol%. The type strain, isolated from a Ghanaian cocoa fermentation in 2004, is LMG 24289^T^ = DSM 21416^T^. The 16S rRNA gene of the type strain has the GenBank/EMBL/DDBJ accession number FM179678.

## Detection and typing of *Weissella*

From 2015 (Fusco et al., 2015) up to date, no advancements have been achieved in the isolation of weissellas: modified CHALMERS (Pepe et al., 2001) and de Man, Rogosa, and Sharpe (MRS) (DeMan et al., 1960) broth enrichment combined with plating on MRS agar added with 2,3,5-triphenyltetrazolium chloride (TTC) (Zamudio-Maya et al., 2008), remain the only media that, among other LAB, allow the differentiation and isolation of weissellas, while *Leuconostoc* selective medium (LUSM) (Benkerroum et al., 1993), sourdough bacteria (SDB) medium (Kline and Sugihara, 1971), and MRS, allow the isolation of weissellas apart from presumptive lactobacilli and Leuconostoc. As for the identification of weissellas, biochemical methods such as those based on the comparison of total soluble cell protein patterns (Dicks, 1995; Tsakalidou et al., 1997) and profiles of cellular fatty acids (Samelis et al., 1998), as well as commercial identification kits such as the Phoenix Automated Microbiology System (Becton Dickinson Diagnostic Systems, Sparks, MD), theVitek2 system (Bio Merieux, Marcy l'Etoile, France), the API50 CHL kit (BioMérieux, Lyon, France) (Lee K. W. et al., 2012), and the RapID™ STR System (Thermo Scientific, Hudson, NH, USA), do not allow an accurate and reliable identification (Fusco et al., 2015; Sturino, 2018). The culture-based identification of weissellas has been improved by matrix-assisted laser desorption–ionization time of flight mass spectrometry (MALDI-TOF MS) (Albesharat et al., 2011; Fairfax et al., 2014; Lee M. R. et al., 2015; Kim et al., 2017, 2021a,b; Nacef et al., 2017; Wang et al., 2020b; Joglekar et al., 2023).

As for the DNA-based taxonomical methods, to overcome the low reliability of 16S rRNA gene sequencing for discriminating highly phylogenetically related weissellas (Kulwichit et al., 2007; Fairfax et al., 2014; Medford et al., 2014; Joglekar et al., 2023), several methods have been developed such as ribotyping (Björkroth et al., 2002), amplified ribosomal DNA restriction analysis (ARDRA) (Jang et al., 2021), denaturing gradient gel electrophoresis (DGGE) of PCR amplified fragments of the 16S rRNA gene (Walter et al., 2001), and sequence typing of *phe*S, *gyr*B, and *dna*A genes, with the *phe*S gene providing the better taxonomic resolution (Joglekar et al., 2023). A genus-specific PCR assay, targeting the 16Sr RNA gene, was developed by Schillinger et al. (2008) for the differentiation of *Weissella* and *Leuconostoc*. Fusco et al. (2011) developed a species-specific PCR for *Weissella confusa* from an AFLP (amplified fragment length polymorphism)-derived marker, whereas a conventional PCR and a real-time PCR were developed by Snyder et al. (2015) for the identification and quantification of *W. ceti* NC36. A real-time PCR assay was developed by Gómez-Rojo et al. (2015) to quantitatively detect *W. viridescens* in blood sausages, whereas following a pan-genome analysis, Kim et al. (2022b) designed species-specific pairs of primers for the real-time PCR detection of 11 *Weissella* species. Finally, Ma et al. (2022) developed an aptasensor based on fluorescence polarization for the detection of *W. viridescens*.

Culture-independent approaches including PCR-DGGE and next-generation sequencing approaches such as metagenetics and metagenomics have allowed the detection of weissellas in various ecological niches (Table 2). Whole-genome sequencing is another approach that is being widely used to identify and characterize weissellas (Benomar et al., 2011; Kim et al., 2011; Amari et al., 2012; Lee J. H. et al., 2012; Figueiredo et al., 2014a,b, 2015; Tanizawa et al., 2014; Malik et al., 2016; Heng et al., 2017; Ku et al., 2017; Li et al., 2017; Du et al., 2018; Garcia-Cancino et al., 2019; Kwak et al., 2019; Panthee et al., 2019; Lin et al., 2020; Månberger et al., 2020; Baugh et al., 2021; Contente et al., 2021; Jang et al., 2021; Patrone et al., 2021; Yuan et al., 2021; Apostolakos et al., 2022; Fanelli et al., 2022; Fukuda and Nolasco-Hipolito, 2022; Surachat et al., 2022; Teixeira et al., 2022).

**Table 2:** Detection of weissellas by culture-independent approaches in various ecological niches.

**Species**	**Origin**	**Country**	**Detection method**	**References**
*Weissella* spp. and *W. paramesenteroides*	Mexican pozol (fermented maize dough)	Mexico	PCR-DGGE (denaturing gradient gel electrophoresis)	Ampe et al., 1999
*W. confusa*	kimchi	Korea	PCR-DGGE	Lee et al., 2005
*W. confusa* and *W. cibaria*	breast milk of healthy women	Spain	PCR-DGGE	Martín et al., 2007
*W. confusa* and *W. viridescens*	Doenjang (fermented soy bean paste)	Korea	PCR-DGGE	Kim et al., 2009
*Weissella* spp., *W. paramesenteroides*	Jeotgal (fermented sea food)	Korea	barcoded pyrosequencing and PCR– DGGE	Roh et al., 2010
*W. hellenica* and *W. paramesenteroides*	Raw milk cheeses	Denmark	Barcoded pyrosequencing of DNA and cDNA	Masoud et al., 2012
*W. cibaria, W. soli*, and *W. koreensis*	Dongchimi (water kimchi)	Korea	Pyrosequencing of 16S rRNA genes	Jeong et al., 2013
*W. hellenica* and *W. paramesenteroides*	Croatian raw ewe's milk cheeses	Croatia	Pyrosequencing of 16S rRNA genes	Fuka et al., 2013
*Weissella* spp., *W. cibaria*, and *W. paramesenteroides*	Nukadoko (naturally fermented rice bran mash used for pickling vegetables	Japan	Pyrosequencing of tagged 16S rRNA gene amplicons	Ono et al., 2014
*Weissella* spp., *W. soli*, and *W. beninensis*	Malt (produced by industrial malting)	Belgium	Culture-independent T-RFLP (terminal restriction fragment length polymorphism) and pyrosequencing	Justé et al., 2014
*Weissella* spp.	oral microbiota of sailors during a long sea voyage	China	Pyrosequencing of 16S rRNA genes Metagenomics	Zheng et al., 2015
*W. cibaria, W. confusa*, and *W. viridescens*	Shanxi aged vinegar	China	Culture -independent PCR-DGGE	Nie et al., 2015
*Weissella* spp*., W. cibaria, W. confusa, W. hellenica*, and *W. viridescens*	Chichi (maize-based fermented beverage)	Spain	Pyrosequencing of 16S rRNA genes	Elizaquível et al., 2015
*Weissella* spp.	Traditional Chinese Yellow Rice Wine	China	Metagenomics	Fang et al., 2015
*Weissella* spp.	Kimchi	Korea	Metagenomics	Jung et al., 2011
*Weissella* spp. *W. confusa*, and *W. cibaria*	Mul kimchi (water radish kimchi)	Korea	Pyrosequencing of 16S rRNA genes	Kim et al., 2012
*Weissella* spp.	Cotija (Mexican ripened cheese)	Mexico	Metagenomics	Escobar-Zepeda et al., 2016
*Weissella* spp. *W. confusa*, and *W. cibaria*	Korean commercial kimchi	Korea	Pyrosequencing of 16S rRNA genes	Kim et al., 2016
*Weissella* spp.	Yucha (traditional Chinese fermented food)	China	Pyrosequencing of 16S rRNA genes	Zhang et al., 2016
*Weissella* spp.	Kimchi	China	Pyrosequencing of 16S rRNA genes	Lee M. et al., 2017
*Weissella* spp.	Soy sauce	China	Pyrosequencing of 16S rRNA genes	Wang H. et al., 2017
*Weissella* spp.	Commercially prepared, domestic and imported, pasteurized (*n =* 8) and unpasteurized (*n =* 7) Gouda cheese	USA	Pyrosequencing of 16S rRNA genes	Salazar et al., 2018
*Weissella* spp.	Zhacai paocai (fermented vegetable)	China	Pyrosequencing of 16S rRNA genes	Liang et al., 2018
*Weissella* spp.	Vaginas of pregnant women	Korea	Pyrosequencing of 16S rRNA genes	You et al., 2019
*Weissella* spp.	Dhanaan (Ethiopian fermented camel milk)	Ethiopia	Pyrosequencing of 16S rRNA genes	Berhe et al., 2019
*Weissella* spp. and *W. viridescens*	high-moisture Mozzarella cheeses	Italy	Pyrosequencing of 16S rRNA genes	Marino et al., 2019
*Weissella* spp.	JIUYAO (fermentation starter traditionally used in Shaoxing-jiu)	China	Barcoded pyrosequencing	Chen et al., 2023
*Weissella* spp.	Stool samples from children with Immunoglobulin A vasculitis and from healthy children	China	Metagenomics	Cao et al., 2021
*W. ghanensis*- group	Fermented cocoa beans	Colombia	Pyrosequencing of 16S rRNA genes	Fernández-Niño et al., 2021
*Weissella* spp.	Fermented potherb mustard (*Brassica juncea* var. *multiceps*),	China	Metagenomics	Liu et al., 2021
*Weissella* spp.	Medium temperature daqu starter	China	Metagenomics	Yang et al., 2021
*Weissella* spp.	Chinese Xiaoqu jiu (liquor)	China	Metagenomics	Zhao C. et al., 2021
*Weissella* spp.	Malaysian naturally fermented silage	Malaysia	Pyrosequencing of 16S rRNA genes	Hisham et al., 2022
*Weissella* spp.	fresh manure from cows, chickens, horses, and pigs	India	Pyrosequencing of 16S rRNA genes	Mutungwazi et al., 2022
*Weissella* spp., *W. soli*	Suancai (Chinese traditional fermented food)	China	Metagenomics	Song et al., 2022
*Weissella* spp. and *W. confusa*	Pickled vegetables	Saudi Arabia	Metagenomics	Yasir et al., 2022
*Weissella* spp.	Fermented mustard	China	Metagenomics	Yu et al., 2022
*Weissella* spp., *W. paramesenteroides*, and *W. cibaria*	Daqu (fermenting agent in Chinese huangjiu and baijiu production)	China	Metagenomics	Zhang et al., 2022
*Weissella* spp.	Daqu (fermenting agent in Chinese huangjiu and baijiu production)	China	Pyrosequencing of 16S rRNA genes	Cheng et al., 2023
*Weissella* spp.	beef, chicken, and pork meat	Malaysia	Pyrosequencing of 16S rRNA genes	Emamjomeh et al., 2023
*W. jogaejeotgali*	Homemade produced herby cheese	Turkey	Pyrosequencing of 16S rRNA genes	Rüstemoglu et al., 2023
*Weissella* spp.	Non-gxiangxing daqu	China	Pyrosequencing of 16S rRNA genes	Xia et al., 2023

## An update on the ecology of *Weissella* and *Periweissella*

*Weissella* and *Periweissella* species may play a role in the fermentation process of products both intended for human and animal consumption. They have been described as components of the fermentative microbiota of crop silages intended as animal feed (Otoni et al., 2018; Dong et al., 2020; Wang et al., 2020a; Wen Fang Wu Wu et al., 2021), where *Weissella* spp. may be generally involved in early stages of fermentation (0–7 days) or even remain relatively stable during the subsequent stages of the process (Wang et al., 2020a). Apart from such products that are not intended for human nutrition, Fusco et al. (2015) reported that *Weissella* and *Periweissella* species are also found in various fermented foods including wheat sourdough, cheeses, fermented meat-, milk-, fish-, and plant-based products (Fusco et al., 2015). However, in the last decade, a growing number of studies are aiming to characterize the microbiota of various fermented foods, helping to increase the awareness that *Weissella* and *Periweissella* may play a role in a wide variety of traditional and novel fermented foods (Table 3).

**Table 3:** Various isolation sources of *Weissella* and *Periweissella* species reported from 2015 up to date.

**Source**	**Sample**	**Species**	**Country**	**References**
**Fermented foods**				
Plant-based fermented foods	*Kimchi* (salted fermented cabbage-based product)	*W. cibaria, W. confusa, W. hellenica, W. koreensis, W. paramesenteroide, W. soli*, and *P. fabaria*	Korea	Lee et al., 2015a; Kim et al., 2017; Yoon et al., 2023
	*Pozol* (beverage obtained from the non-alcoholic fermentation of nixtamalized (lime-cooked) maize)	*W. cibaria, W. confusa, W. paramesenteroides*, and *Weissella* spp.	Mexico	López-Hernández et al., 2018; Hernández-Oaxaca et al., 2021
	*Soidon* (spontaneously fermented bamboo shoots)	*W. cibaria, W. oryzae*, and *P. ghanensis*	India	Romi et al., 2015
	Spontaneously fermented sorghum sourdough	*W. cibaria, W. confusa*, and *W. paramesenteroides*	Italy	Falasconi et al., 2020
	Spontaneously fermented ginger pickles	*W. cibaria, W. confusa*, and *P. fabaria*	China	Xiang et al., 2022
	Spontaneously fermented maize brans	*W. cibaria* and *W. confusa*	Italy	Decimo et al., 2017
	Spontaneously fermented faba bean (*Vicia faba major* and *V. faba minor*) sourdoughs	*W. cibaria* and *W. koreensis*	Italy	Coda et al., 2017
	Fermented pineapple peel-derived beverage	*W. paramesenteroides* and *P. ghanensis*	Indonesia	Tallei et al., 2022
	Spontaneously fermented rye dough	*W. cibaria*	Spain	Llamas-Arriba et al., 2021; Hernández-Alcántara et al., 2022
	Spontaneously fermented chia (*Salvia hispanica* L.) sourdough	*W. cibaria*	Argentina	Dentice Maidana et al., 2020
	Spontaneously fermented chickpea (*Cicer arietinum*) sourdough	*W. confusa*	Italy	Galli et al., 2020b
	Homemade fermented soybean product	*W. confusa*	Indonesia	Heng et al., 2017
	Table olives	*W. paramesenteroides*	Morocco	El Issaoui et al., 2021
	*Nukadoko* (fermented rice bran)	*W. soli*	Japan	Fukuda and Nolasco-Hipolito, 2022
	*Kocho* (fermented Enset plant (*Ensete ventricosum* (Welw.) Chees man, Musaceae)	*P. beninensis*	Ethiopia	Andeta et al., 2018
	Spontaneously fermented cocoa beans	*P. fabalis*	Nicaragua	Papalexandratou et al., 2019
	Fermented pickled cowpea (*Vigna unguiculata* [Linn.] Walp)	*Weissella* spp.	China	Guo et al., 2021
Meat-based fermented foods	*Pirot* “ironed” sausage (fermented meat with spices)	*W. cibaria, W. koreensis*, and *P. fabalis*	Serbia	Bogdanović et al., 2023
	*Calabresa* (fermented meat product)	*W. viridescens*	Brazil	Castilho et al., 2019
Seafood-based fermented foods	*Gajami-sikhae* (salted fermented fish mixed with vegetables and millet)	*W. cibaria, W. hellenica, W. kandleri, W. koreensis*, and *W. viridescens*	Korea	Kim et al., 2022a
	*Jeotgal* (salted fermented seafood)	*W. halotolerans* and *W. thailandensis*	Korea	Kim et al., 2017
	*Kung-som* (fermented shrimp)	*W. thailandensis*	Thailand	Saelao et al., 2016
Milk-based fermented foods	Spontaneously fermented thermized cow milk	*W. confusa*	Malaysia	Goh and Philip, 2015
	*Lait caillé* (spontaneously fermented raw cow milk product)	*W. paramesenteroides*	Burkina Faso	Bayili et al., 2019
Insect-based fermented foods	Spontaneously fermented cricket powder	*W. confusa*	Italy	Galli et al., 2020a
**Non-fermented foods**				
Dry-cured or pickled foods	*El-Guedid* (dry-cured meat product)	*W. cibaria, W. confusa, W. hellenica, W. paramesenteroides, W. thailandensis*, and *W. viridescens*	Algeria	Bader et al., 2021
	*Pastirma* (dry-cured meat product)	*W. cibaria, W. confusa, W. halotolerans*, and *W. hellenica*	Turkey	Öz et al., 2017
	pickled white cabbage	*W. cibaria, W. confusa*, and *W. soli*	Reunion Island (Africa)	Fessard and Remize, 2019
**Fermented products not intended for human consumption**				
Silages	Silages of alfalfa (*Medicago sativa* L.), whole-plant corn (*Zea mays* L.), and their mixture	*W. cibaria, W. confusa, W. koreensis*, and *Weissella* spp.	China	Wang et al., 2020a
	Guinea grass (*Panicum maximum* Jacq. cultivar Mombasa) silages	*W. confusa, W. oryzae*, and *W. paramesenteroides*	Brazil	Otoni et al., 2018
	Pineapple peel silage	*P. ghanensis*	Costa Rica	Wen Fang Wu Wu et al., 2021
	Ensiled sweet sorghum (*Sorghum bicolor* (L.) Moench) bagasse	*Weissella* spp.	China	Dong et al., 2020
**Raw fruits and vegetables**				
Raw fruits	Tomato (*W. cibaria*), papaya (*W. confusa, W. paramesenteroides*)	*W. cibaria, W. confusa*, and *W. paramesenteroides*	Reunion Island (Africa)	Fessard and Remize, 2019
	Custard apple (*W. cibaria*), Guava (*W. minor*), Khaki (*P. fabalis*)	*W. cibaria, W. minor*, and *P. fabalis*	Argentina	Ruiz Rodríguez et al., 2019
	Banana (*Musa* spp.) fruits	*W. cibaria* and *W. paramesenteroides*	Taiwan	Chen et al., 2017
	Native fruit of Ecuadorian Amazon	*W. confusa*	Ecuador	Garzón et al., 2017
	Various fresh fruits (sapota, cherry, banana, orange, plum)	*W. paramesenteroides*	Not Reported	Pabari et al., 2020
Raw vegetables	Garlic (*Allium sativum*), ginger (*Zingiber officinale*), Korean leek (*Allium tuberosum*)	*W. cibaria*	Korea	Lee et al., 2015a
**Humans**				
Intestine	Feces from healthy women	*W. confusa*	China	Wang et al., 2020b
	Feces of young healthy child	*W. confusa*	South Korea	Jin et al., 2019
	Stool samples of adults	*Weissella* spp.	China	Zhang et al., 2022a
Oral cavity	Saliva of an infant	*W. cibaria*	South Korea	Kang et al., 2017
	Saliva of 3–5 years old children with or without caries	*Weissella* spp.	China	Wu et al., 2023
	Oral samples collected immediately after birth of full-term vaginally delivered newborns	*Weissella* spp.	Not Reported	Singh et al., 2020
Vagina	Vaginal swabs from pregnant women	*Weissella* spp.	Korea	You et al., 2019
Milk	Milk from healthy nursing mothers (full-term pregnancy; 10 days−10 months postpartum)	*W. confusa*	Brazil	Reis et al., 2016
	Milk from mother (cesarean delivery; lactating period between 21-48 weeks) suffering from asthma and overweight	*Weissella* spp.	Spain	Marin-Gómez et al., 2020
**Animals**				
Intestine of mammals	Feces of Nili-Ravi Buffalo (*Bubalus bubalis*)	*W. cibaria, W. confusa, W. bombi*, and *W. soli*	Pakistan	Khalil et al., 2022
	Fecal samples of European badgers (*Meles meles*)	*W. cibaria* and *W. paramesenteroides*	United Kingdom	Stedman et al., 2018
	Feces of giant panda (*Ailuropoda melanoleuca*)	*W. cibaria* and *Weissella* spp.	China	Zhao S. et al., 2021
	Feces of camels	*W. confusa and W. halotolerans*	Tunisia	Fhoula et al., 2018
	Feces of giant panda (*Ailuropoda melanoleuca*)	*W. cibaria*	China	Du et al., 2018
	Droppings of captive Saki monkey	*W. cibaria*	France	Eveno et al., 2021
	Fecal samples of Gannan yaks	*W. cibaria*	China	Zhang et al., 2022b
	Fecal samples of cows	*W. cibaria*	Kuwait	Patrone et al., 2021
Intestine of fish	Freshwater fish (*Cirrhinus mrigala*) intestine	*W. cibaria*	India	Govindaraj et al., 2021
	Gut of rainbow trouts (*Oncorhynchus mykiss* Walbaum)	*W. oryzae*	Iran	Mortezaei et al., 2020
	Intestinal tracts of tiger pufferfish (*Takifugu rubripes*)	*Weissella* spp.	China	Gao et al., 2022
	Digesta from the middle intestine of grass carp (*Ctenopharyngodon idella*)	*Weissella* spp.	China	Yang G. et al., 2022
Intestine of birds	Broiler chicken feces	*W. cibaria*	Not reported	García-Hernández et al., 2016
Intestine of insects and slime of molluscs	Gut of ants (*Cataglyphis*)	*W. halotolerans*	Tunisia	Fhoula et al., 2018
	Intestinal content of *Locusta migratoria manilensis* (Meyen)	*Weissella* spp.	China	Wang W. et al., 2022
	Slime of garden snail (*Helix aspersa* Müller)	*W. viridescens*	Not reported	Garcia-Cancino et al., 2019
Rumen	Rumen liquid of Holstein lactating cows	*Weissella* spp.	United Kingdom	Stergiadis et al., 2021
Vagina	Vaginal swabs from postpartum dairy cows	*W. confusa* and *W. koreensis*	China	Zhao et al., 2015; Wang et al., 2016
Milk	Buffalo milk	*W. confusa, W. hellenica*, and *W. paramesenteroides*	Brazil	Tulini et al., 2016
	Raw goat milk	*W. cibaria* and *W. confusa*	Nigeria	Akinyemi et al., 2022
	Camel raw milk	*W. cibaria* and *W. confusa*	Morocco	Mercha et al., 2020
	Dromedary raw milk	*W. cibaria*	Iran	Davati et al., 2015
	Camel fresh milk	*W. confusa*	Mongolia	Zhao et al., 2019
	Raw cow milk	*W. paramesenteroides*	Maltese Islands (Europe)	Garroni et al., 2020

The increasingly popular and worldwide consumed *kimchi*, a salted fermented cabbage-based Korean food (Lee et al., 2022), is a well-known source of *W. cibaria, W. confusa, W. koreensis, W. hellenica, W. paramesenteroides, W. soli*, and *P. fabaria* (Lee et al., 2015a; Kim et al., 2017; Yoon et al., 2023, Table 3). Moreover, *Weissella* and *Periweissella* spp. are also being reported as part of the inhabiting microbiota also in other lesser-known traditional fermented products (Table 3). For instance, *W. paramesenteroides* was detected during the production of *lait caillé*, a spontaneously fermented traditional raw milk product made in Burkina Faso (Bayili et al., 2019). In particular, Bayili et al. (2019) found that *W. paramesenteroides* was more abundant during the early stages of fermentations (0–7 h), while it could no longer be recovered later, until the end of fermentation (59 h) (Bayili et al., 2019). The presence of *Weissella* is also reported in novel fermented products that are being developed to meet the growing human dietary needs. Galli et al. (2020a) recently detected *W. confusa* (along with other LAB belonging to *Latilactobacillus, Lactiplantibacillus, Lactococcus*, and *Enterococcus* genera) during the spontaneous fermentation of a cricket powder, which was propagated through a backslopping procedure. Coda et al. (2017) reported the presence of *Weissella* during the spontaneous backslop-propagated fermentation of two faba bean (*Vicia faba minor and Vicia faba major*) flours that could be used as substitutes of animal-derived protein sources. It was found that, among LAB, *W. koreensis* showed one of the highest incidences of occurrence in the tested faba bean sourdoughs, although also the presence of *W. cibaria* was reported during the fermentation of these sourdough samples (Coda et al., 2017). Furthermore, Decimo et al. (2017) found *W. cibaria* and/or *W. confusa* during the initial stages of spontaneous fermentation of two types of commercial native maize brans, which could potentially be exploited as functional food in human nutrition. Apart from legume- and cereal-derived fermented products, *Weissella* species may also be found during the fermentation of oilseed- and other naturally gluten-free cereal-derived products, representing alternative foods for people with celiac disease (Falasconi et al., 2020). In particular, *W. cibaria* was detected during the spontaneous fermentation of chia (*Salvia hispanica* L.) sourdough, being found as one of the dominant species at the final stages (8–10 days) of a backslopping-propagated fermentation (Dentice Maidana et al., 2020), while *W. paramesenteroides, W. confusa*, and *W. cibaria* were detected at the early stages (0–1 days) of the spontaneous fermentation of a sorghum sourdough, similarly obtained using a backslopping procedure (Falasconi et al., 2020).

Therefore, as other LAB, *Weissella* and *Periweissella* species often participate during the fermentation process of various products owing to their enzymatic capabilities that are particularly adapted toward carbohydrate metabolism, as was recently reported (Hernández-Oaxaca et al., 2021; Fanelli et al., 2022). Nevertheless, the dynamics and microbial successions during the fermentation process are likely to be influenced by (i) the initial microbial composition of the raw materials, (ii) the physico-chemical composition of the products to be fermented, (iii) the fermentation conditions (e.g., temperature and oxygen availability) and procedures applied, and (iv) the metabolic interactions with other microorganisms constituting the microbiota. Additionally, *Weissella* species have been also detected in some pickle- and dry-cured products (Table 3), necessitating more targeted studies to better understand and describe the role of *Weissella* and *Periweissella* within the fermentative and curing processes that to date remains not yet fully clarified.

*Weissella* and *Periweissella* spp. can also be associated with raw foods (Table 3), including animal and human milk.

In particular, apart from human-derived milk (Oikonomou et al., 2020; Mantziari and Rautava, 2021), and beyond the detection of *Weissella* spp. in milk of commonly raised dairy animals such as cows, ewes, and goats, as well as milk of companion animals, such as dogs (Fusco et al., 2015), *Weissella* is being also reported in milk of less common domesticated animals. In particular, although enterococci were the most frequently isolated LAB, *W. cibaria* was found in the raw milk from dromedary in Iran (Davati et al., 2015), while *W. confusa*, either alone or together with *W. cibaria*, was isolated from raw camel milk in Mongolia and Morocco (Zhao et al., 2019; Mercha et al., 2020). Although, to the best of our knowledge, the ecology and the relevant sources of *Weissella* species in milk have not been clearly elucidated, various mechanisms are hypothesized to shape the milk microbiota both in humans and animals. Apart from the mere contamination of milk immediately after excretion, due to the presence of microorganisms that inhabit the skin or originate from fecal or environment contamination, three other mechanisms may represent possible sources of the milk microbiota, as was suggested for milk from both humans and cows (Oikonomou et al., 2020; Mantziari and Rautava, 2021). These include the (i) enteromammary pathway through which dendritic cells or macrophages transfer bacteria from the maternal gut to the mammary gland and then release the bacteria in the milk, (ii) the retrograde backflow of bacteria from the skin, the environment, or the offspring oral cavity during suckling or milking, and (iii) the presence of a resident microbiota in the mammary tissue (Oikonomou et al., 2020; Mantziari and Rautava, 2021). *Weissella* species, that are known to occur in different areas of the human and animal body including rumen and vagina (Table 3), are also found in the oral cavity of young children, as well as in human and animal intestine (detected mainly by fecal sampling) (Table 3); therefore, these may represent possible starting sites for *Weissella* to reach the human and animal milk, needing further investigations.

Notably, *Weissella* spp. can inhabit the intestinal tract of both vertebrates and invertebrates, the latter including insects and molluscs (Table 3), where they may be associated with the healthy status of the gut. In this regard, a recent study conducted on the feces of giant pandas found in healthy sub-adult animals a higher abundance of *Weissella* spp., including *W. cibaria*, when compared to sub-adult animals suffering from anorexia (Zhao S. et al., 2021). Interestingly, a symbiotic relationship among *Weissella* and *Leuconostoc, Bacillus*, and *Streptococcus* genera appeared to occur in the gut of the analyzed subjects, and, conversely, a decrease in *Weissella* and *Streptococcus* and increase in *Clostridium* could be the cause of the reported anorexia symptoms (Zhao S. et al., 2021).

*Weissella* and *Periweissella* may thus inhabit various ecological niches, with *W. cibaria, W. confusa*, and *W. paramesenteroides* being frequently reported in different sources (Table 3). Less knowledge is available for other species, especially those lastly described (*W. coleopterorum, P. cryptocerci, W. muntiaci, W. fangxianensis*, and *W. sagaensis*) for which, apart from the studies that recently reported their primary source of isolation (Table 1), no further knowledge is substantially available. This, therefore, deserves future investigations to clearly identify their relevant niches and better understand the ecological role also of the novel *Weissella* and *Periweissella* species.

## An update of the technological potential of *Weissella* and *Periweissella*

**Figure 1:**
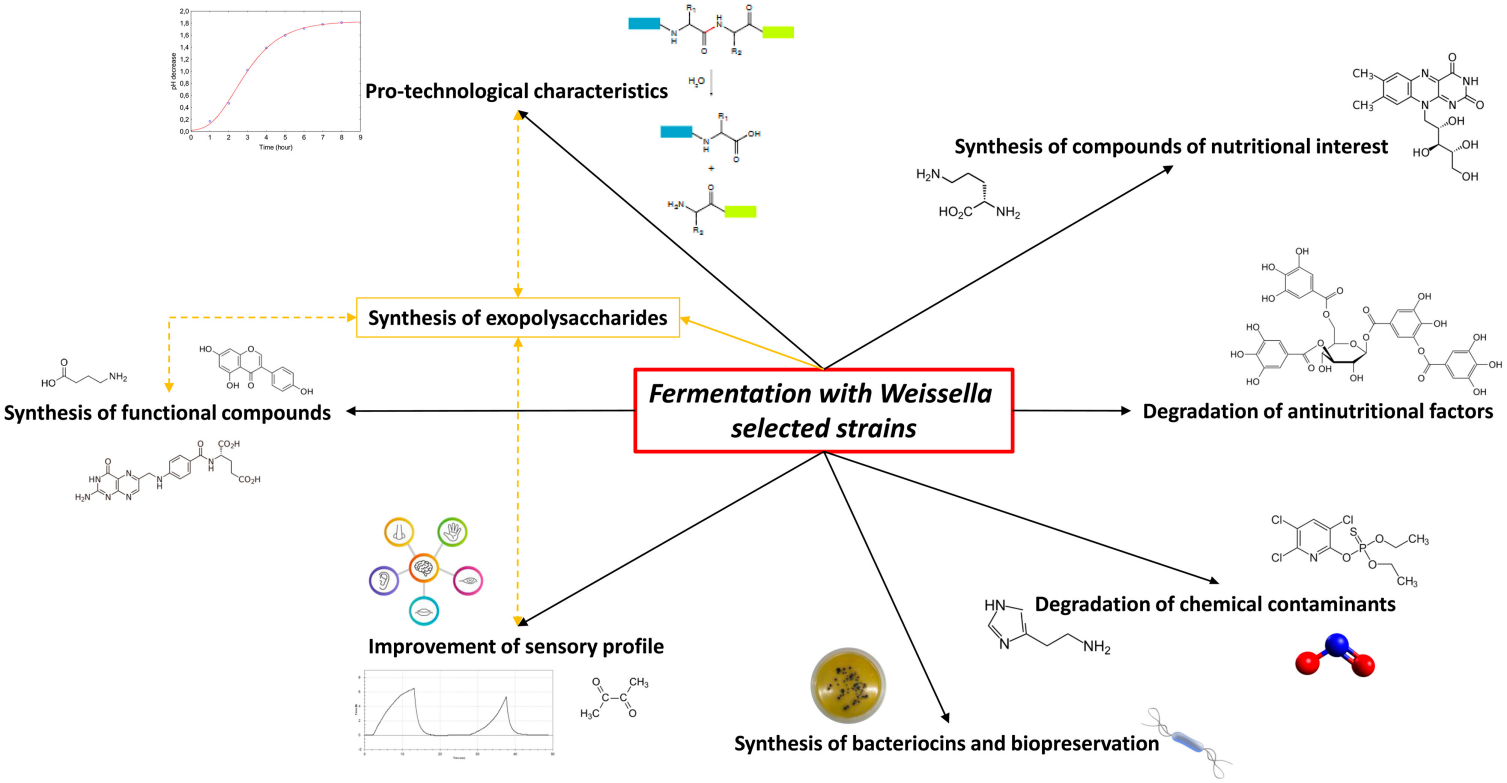
Potential of *Weissella* spp. selected strains as starters in food fermentation applications. The yellow lines indicate the correlation between the exopolysaccharides production by *Weissella* spp. and the improvement of food characteristics.

Due to a long history of safety and the optimal pro-technological characteristics, LAB can be considered as the most important microbial group acting as starters in traditional and novel fermented foods and many of their functions have long been investigated and understood. Among these, *Weissella* spp. strains own numerous technological and functional properties and frequently play a significant part in food preservation and health benefits; thus, they have been evaluated as innovative starter cultures with an industrially significant interest (Fessard and Remize, 2017). However, their use as starters for food and beverage fermentation such as their inclusion in commercial products is still limited due to the lack of safety evaluation by two major food safety authorities, namely, the Food and Drug Administration (FDA) and the European Food Safety Authority (EFSA), which still do not consider any strain belonging to this genus as GRAS or QPS, respectively. A paucity of scientific data evaluating the safety aspects, antibiotic resistance pattern, potential biogenic amine synthesis, and infection risk partly explains such neglecting (Fessard and Remize, 2017; Ahmed et al., 2022). In the last years, weissellas characterization including technological and functionality assessments was widely performed to demonstrate also the health effects of single strains in accordance to the definition of probiotics by the International Scientific Association for Probiotics and Prebiotic (ISAPP). Therefore, the weissellas isolates from different fermented products have been characterized as potential starters to be used in food processing (Fusco et al., 2015), increasing their possible applications (Figure 1).

### Selected strains for food fermentation

The suitability of *W. cibaria* and *W. confusa* to be used as starters for bread making was analyzed following an integrated approach including phenotypic, genotypic, and metabolomic characteristics. *W. cibaria-com2* was identified (Lopez et al., 2022) as a strain able to perform an intense proteolysis in wheat flour doughs leading to release huge amounts of numerous amino acids and peptides, some of which belong to the branched-chain amino acid-derived compounds (BCAA). Thus, the differential metabolite profile of that strain was partially explained by a genome investigation for putative differences in peptidases, proteases, and amino acid/peptide transporters (Lopez et al., 2022). In particular, a higher presence of amino acid permeases, peptidases (C40 family), and oligopeptide ABC transporters was found in this strain as compared to other *Weissella* spp. strains (Lopez et al., 2022). Proteolysis is indeed considered as a key process in food fermentation leading toward a higher impact on the technological, nutritional, organoleptic, and functional features of the fermented foods.

*W. confusa* SD8 was used for making a sorghum sourdough in the study of Olojede et al. (2022) and was found to confer an optimal specific volume to the bread and moreover also contributed to tannin degradation (Olojede et al., 2022). Tannins are very abundant in sorghum flour (such as in other minor cereals, pseudocereals, and legumes) and considered as antinutritional factor since they are able to bind proteins making them refractory to digestion and are responsible for the bitter taste of different plant-based ingredients.

A *W. koreensis* strain (DB1) isolated from *kimchi* producing high levels of ornithine was proposed as a functional starter culture for rice bran fermentation (Yeong et al., 2020). Ornithine is produced in microbes from arginine throughout the intracellular arginine deiminase pathway (Yeong et al., 2020). Different *Weissella* strains were also proposed as starters for *kimchi* production due to their technological characteristics, even though these were also found to exhibit a weak hemolytic activity (Jeong and Lee, 2015). *W. cibaria* M3 was used (as mixed starter, in association with *Lactococcus lactis*) to produce a typical Chinese fermented fish product (*Chouguiyu*), showing optimal organoleptic characteristics (Bao et al., 2018). A folate-producing strain of *W. cibaria* (PL17-3) was also selected for the production of fermented fish (Deatraksa et al., 2018). Sixteen *Weissella* isolates from artisanal Brazilian cheeses were identified as potential starter candidates for the dairy industry owed to their high acidification ability, diacetyl production, and proteolytic activity (Teixeira et al., 2021). A selected *W. cibaria* strain was demonstrated to improve the organoleptic profile of *Sichuan pickle* when used in association with *Lactiplantibacillus plantarum* (Xiang et al., 2020).

*W. cibaria* 30 and *W. cibaria* 64, isolated from tropical fruits, were included in a list of starters for fruits and vegetables fermentation, whereas *W. soli* 58 showed the characteristics of a potential preservative culture for fruits and vegetables (Fessard and Remize, 2019). *W. cibaria* FB069 was tested as a starter to produce functional fermented soymilk. The addition of xylooligosaccharides before the fermentation process led to considerable increase of the acidification rate, viscosity, and *W. cibaria* FB069 growth. Moreover, the synbiotic fermented product obtained was characterized by increased levels of dextran, folate, GABA, genistein, and daidzein, and it was able to decrease the proliferation of Caco-2 and HCT116 cell lines (Le et al., 2020).

A spontaneous mutant strain, *W. cibaria* BAL3C-5 B2, was selected among different parental and mutant strains of *W. cibaria* to produce a content riboflavin bread, characterized by concentrations over 0.1 mg of riboflavin in 100 g of bread (Hernández-Alcántara et al., 2022). Moreover, the use of *Weissella* strains was also demonstrated in the production of baker's yeast-free bread (Lopez et al., 2022).

### The importance of exopolysaccharides (EPSs)

LAB able to produce EPS play a pivotal role in industry for the development of functional food and are also used as coadjutants or starter cultures for the development of yogurt and other traditional fermented foods (Zhu et al., 2018). In these products, the EPS production by LAB starter cultures may occur *in situ*. As a consequence, since LAB EPS improve the texture and rheology of fermented foods by acting as natural biothickeners, the use of food additives, such as pectin and starch, may be avoided (Zhu et al., 2018). Furthermore, LAB EPS may act as probiotic and prebiotic due to their immunoregulatory, antitumoral, and antioxidant activities, as well as cholesterol-lowering ability, and prebiotic effects (Zhu et al., 2018) (for the probiotic and prebiotic role of the weissellas' EPS, see the subheader “*Exopolysaccharides produced by weissellas”* in the paragraph on the probiotic potential below). Among LAB, weissellas can generate high content of EPS without excessive amount of acetate which represents undesired compound in some products (e.g., beverages) also in the presence of added sugar usually used to maximize the EPS production. Indeed, the addition of sucrose during weissellas fermentation leads to dextran production and the use of fructose as a carbon source and not as electron acceptor, thus generating minimal quantities of acetate instead of mannitol (Rolim et al., 2019).

*Weissella confusa* A16, a strain characterized by a high level synthesis of dextran, was efficiently used for the EPS enrichment of a brewer's spent grain added with 4% of sucrose, aimed at improving its technological properties and contributing to its recycle as food ingredient (Koirala et al., 2021). As natural structure-forming agent, dextran has already been used as a food additive. Its supplementation to food formulations efficiently improved the technological properties of different raw materials such as fiber- and protein-rich matrices (such as cereal by-products), allowing their use as ingredients in food production chains (Koirala et al., 2021). Viscosity increase in fermented brewers' spent grain supplemented with sucrose occurred first after 10 h of fermentation and increased until 24 h concomitantly with dextran accumulation. The dextran content after 24 h was ~1% on the total weight of the brewers' spent grain (Koirala et al., 2021). A dextransucrase gene was identified in *W. confusa* A16 that showed a typical inducible characteristic, with an intense upregulation occurring at 10 h. EPS produced by a strain isolated from distiller grains of *Chinese Baijiu*, namely, *Weissella cibaria* NC516.11, was proven to be able to promote the cross-linking of starch molecules, thus increasing the water-holding capacity (Li et al., 2022). Dynamic rheology indicated that the aqueous solutions of EPS are pseudoplastic fluids, and their addition to gluten-free ingredients increases the viscoelastic features of the dough (Li et al., 2022). The use of EPS producing strain in bread making was extensively exploited in both gluten-free and gluten-containing bakery products. *Weissella cibaria* P9 was used to produce gluten-free bread also including sucrose-containing flour instead of sugar as a precursor (Montemurro et al., 2021, 2023). Moreover, the *in situ* production of EPS by weissellas for improving the steamed bread quality, usually associated with increase of the specific volume, enhance of the texture, and decrease of the staling rate, was recently confirmed (Xu et al., 2020; Sha et al., 2023).

A wholemeal quinoa beverage was also fermented with *W. cibaria* MG1 with the aim to produce a yogurt-like product that showed high water-holding capacity, viscosity, and EPS concentration as a consequence of the fermentation (Zannini et al., 2018). It was hypothesized that the high EPS (dextran) concentration was responsible for the optimal structural properties of the fermented matrix (Zannini et al., 2018).

Other plant-derived substrates were also efficiently *in situ* enriched with EPS using selected strains of *Weissella* spp., such as faba bean flour (Xu et al., 2017; Rizzello et al., 2019), chickpea (Galli et al., 2020a), and rye (Kajala et al., 2016). A *W. cibaria* strain (SJ14) isolated from *Sichuan paocai* (a type of Chinese pickles), characterized by strong salt tolerance, acidification, and nitrite depletion capacities, was identified as a heteropolysaccharide producer. Moreover, a strong antioxidant activity of its EPS was demonstrated (Zhu et al., 2018).

The potential probiotic and exopolysaccharide-producing strain *W. confusa* VP30 was isolated from young children's feces, and its EPS was characterized and quantified. Moreover, the safety was assessed with the aim of applying the strain in food production (Jin et al., 2019). Lastly, a wild *W. minor* (W4451) strain was demonstrated to be able to significantly increase milk viscosity and was therefore proposed as starter for the dairy sector (Bancalari et al., 2020).

### Antimicrobial activity and control of food-associated pathogens

For the antimicrobial activity of weissellas and periweissellas in detail, see the subheader “*Antimicrobial activity of weissellas”* in the paragraph on the probiotic potential below.

The bacteriocin weissellicin D was produced by the strain *W. hellenica* D1501 in fermented pork and showed antimicrobial activity against *Staphylococcus aureus, Listeria monocytogenes*, and *E. coli* (Chen et al., 2014a). *W. hellenica* D1501 was therefore also tested as starter to produce a tofu with long shelf life (Chen et al., 2014b). *W. cibaria* D30 was used in cottage cheese after whey separation from the curd and not only demonstrated inhibitory activity against *L. monocytogenes* ATCC 15313 but also increased the antioxidant properties of the product (Kariyawasam et al., 2019).

One of a possible solution for overcoming the still not authorized use of weissellas in food production is the use of cell-free suspension (CFS), as reviewed by Aggarwal et al. (2022) and Ahmed et al. (2022). CFS is considered postbiotics according to the definition of ISAPP, describing them as a “preparation of inanimate microorganisms and/or their components that confer a health benefit on the host.” *Weissella cibaria* CMU, an oral care probiotic, was discovered to produce and release secreted proteins, organic acid, and hydrogen peroxide with antibacterial activity against periodontal pathogens (Lim et al., 2018). The CFS from *W. viridescens* WV20-15 was tested, excluding the effect of organic acids and hydrogen peroxide, to control *Listeria monocytogenes* 10403S. Inhibitory compounds of proteinaceous nature, probably bacteriocins, decreased the production of microbial biofilm and eradicated preformed biofilms on different materials. Moreover, a significant reduction of *L. monocytogenes* 10403S growth was found on chilled pork (Yang C. et al., 2022). The partially purified bacteriocin 7293 obtained from *W. hellenica* BCC 7293 CFS was effectively used to produce an antimicrobial biodegradable food packaging applied in PLA/SP film. *In vitro* assays demonstrated the inhibition of both Gram-positive (*Staphylococcus aureus* and *Listeria monocytogenes*) and Gram-negative bacteria (*Escherichia coli, Salmonella enterica* serovar Typhimurium, *Pseudomonas aeruginosa*, and *Aeromonas hydrophila*). Moreover, the innovative packaging was used to avoid the proliferation of the pathogenic microorganisms in a challenge test of inhibition of chilled pangasius filet (Woraprayote et al., 2018).

### Reduction of chemical contaminants

Hamoud and Sifour (2021) demonstrated that the potentially probiotic strain *Weissella confusa* Lb. Con was able to survive in MRS broth at a concentration of 200 μg/ml of chlorpyrifos, being able also to degrade about 25% of this pesticide. Considering the wide use of this pesticide to control foliar insects in different vegetables, these results suggested the potential use of this strain in the decontamination of food matrices or in probiotic formulations, aiming at the *in vivo* reduction of pesticide toxicity. Liu et al. (2020) tested *W. cibaria* X31 and *W. confusa* L2 as low nitrite dry-fermented sausages starters. The final product was characterized by high growth rate of both inoculated microorganisms, high degree of redness, high proteolysis rate, and decreased residual nitrites and *S. enterica* growth. Fermented meat can contain not only nitrite but also high amounts of biogenic amines. The use of both *Lactiplantibacillus plantarum* His6 and *Weissella viridescens* F2 as starters for Roucha production led to the decrease of histamine and tyramine of ~50%, due to their amine oxidase activity and the conversion into aldehyde, hydrogen peroxide, and ammonia (Han J. et al., 2022).

## Pathogenic potential of *Weissella* and *Periweissella*

In 2015, Fusco et al. (2015) reviewed all the cases of clinical infections with weissellas that occurred until that year. From 2015 to date, further cases have occurred, all involving *W. confusa* strains (Table 4). As for *Weissella* infections in animals from 2015 to date, only five cases have been reported, with *W. ceti* as the etiological agent of weissellosis in rainbow trouts (Castrejón-Nájera et al., 2018; Mitomi et al., 2018; Medina et al., 2020; Vásquez-Machado et al., 2020).

**Table 4:** *Weissella* infections in humans from 2015 up to date.

**Age and sex**	**Clinical Infection**	**Causative agent**	**Underlying conditions**	**Survival**	**References**
63, F	Bacteremia	*W. confusa*	Multiple abdominal surgeries, central catheter	Yes	Vasquez et al., 2015
78, M	Meningitis	*W. confusa*	Alzheimer's disease, diabetes mellitus type 2, coronary artery disease status	Yes	Cheaito et al., 2020
25, M	Bacteremia	*W. confusa*	Crohn's disease, intestinal failure, short bowel syndrome, history of frequent blood stream infections	No	Spiegelhauer et al., 2020
57, F	Bacteremia	*W. confu*sa	Ulcerative colitis and autoimmune hepatitis and primary sclerosing cholangitis (PSC) overlap requiring liver transplant.	No	Kelkar et al., 2021
63, M	Endocarditis	*W. confusa*	Type 2 diabetes mellitus, hypertension, hypercholesterolemia, and a congenital bicuspid aortic valve	Yes	Hurt et al., 2021
65, M	Endocarditis	*W. confusa*	Alcohol associated cirrhosis, Child-Pugh Classification C, a MELD-sodium score of 18, and liver transplant evaluation	No	Wijarnpreecha and Fontana, 2022
11, M	Septicemia	*W. confusa*	Acute pancreatitis and acute respiratory distress syndrome (ARDS)	Yes	Azim et al., 2023
92, F	Infective endocarditis of a bio-prosthetic valve	*W. confusa*	9 mm Edwards Magna pericardial bovine aortic valve (AV) prosthetic implantation (Edwards Lifesciences, Irvine, CA) and a 25 mm porcine St. Jude bio-prosthetic mitral valve (MV) replacement (St. Jude Medical, Inc., St Paul, MN) in 2014, heart failure with persevered ejection fracture (HFpEF), paroxysmal atrial fibrillation on apixaban, chronic kidney disease (CKD) stage 3B, hypothyroidism, and prior lumbar fusion presented in the fall of 2021 due a two-week history of generalized weakness, dyspnea at rest, and intermittent dark stools	Yes	Massasati and Waseem, 2023

No studies have been published so far about the pathogenic potential of *Periweissella* species.

## Safety assessment of *Weissella* and *Periweissella*

Since 2015, when Fusco et al. (2015) provided an overview of studies published until that year on the probiotic potential of weissellas, many further articles on the same topic have been published up to date. As reported in Tables 5, 6, most of the studies focused on *W. confusa* and *W. cibaria* strains isolated from various ecological niches. For the majority of the potentially probiotic strains, a safety assessment consisting of investigations into the antibiotic susceptibility and the hemolytic activity was performed. However, Sturino (2018) carried out a literature-based safety assessment of *W. confusa*, concluding that many strains of this species can be safely used for poultry in direct-fed microbial products. Cupi and Elvig-Jørgensen (2019) assessed the toxicological safety of *W. confusa* by *in vivo, in vitro*, and *ex vivo* studies. In the tested conditions, no toxic effects were shown by *W. confusa* allowing to conclude that this species could be used as a safe direct-fed microbial product (Cupi and Elvig-Jørgensen, 2019). However, for their studies, they used “an off powder of freeze-dried bacteria composed of almost entirely *W. confusa*,” but no specification on the composition in strain/strains of this powder was made. By contrast, Bourdichon et al. (2021) used 46 strains of *W. confusa* (17 of clinical and 26 of food origin) to provide a safety assessment based on their hemolytic activity and antibiotic susceptibility, as well as on the search of antibiotic resistance genes, virulence determinants, and genes coding for deleterious metabolites (such as biogenic amines) within their genomes. Moreover, a literature search was conducted to find reports of infection caused by strains of *W. confusa* (Bourdichon et al., 2021). This study allowed Bourdichon et al. (2021) to consider *W. confusa* as “safe for use in the food chain, food culture for fermentation, or as probiotic strain candidate”.

**Table 5:** *In vitro* probiotic potential and safety assessment of weissellas and periweissellas.

**Species**	**Strain**	**Source**	**Nation**	**Probiotic potential**	**Safety assessment**	**References**
*W. koreensis*	FKI21	South Indian fermented koozh	India	Survival under simulated human gastro-intestinal tract (hGIt) conditions, antimicrobial spectrum, deconjugation of sodium glycocholate and sodium taurocholate, aggregation activity, exopolysaccharide (EPS) production, *in vitro* cholesterol reduction assay, scanning electron microscopy (SEM)	Antibiotic resistance profile	Anandharaj et al., 2015
*W. cibaria*	CMU	saliva of healthy Korean children	Korea	Resistance again lysozyme, and hydrogen peroxide, acidogenic potential, inhibition of biofilm formation, coaggregation, antibacterial activity against dental caries bacteria, and inhibition of volatile sulfur compounds	-	Jang et al., 2016
*W. cibaria*	Not specified	Not specified	Korea	Anti-inflammatory effect on macrophages of butanol extracts of *Asparagus cochinchinensis* fermented with *W. cibaria*	-	Lee H. A. et al., 2017
*W. cibaria*	WD2	Fermented cassava and wara	Nigeria	Survival under hGIt conditions, tolerance to cadmium and lead, antioxidative activity	Hemolytic activity	Ojekunle et al., 2017
*W. cibaria*	CIATEJ B1-48.1	Tejuino (fermented beverage)	Mexico	Survival under hGIt conditions, antimicrobial spectrum, *in vitro* adhesion capacity, short-chain fatty acids analysis	-	Silva et al., 2017
*W. cibaria* *W. viridescens*	FB-069 FB-077	Salted squid	Korea	Antioxidative activity, antimicrobial spectrum, survival under hGIt conditions, *in vitro* adhesion assay, auto-aggregation and co-aggregation capacity. Salted squid fermentation was carried out using the two probiotic *Weissella* strains and antioxidant capacity of fermented squid samples was determined.	*In vitro* hemolytic reaction, mucin degradation and biogenic amine production. Antibiotic resistance profile	Le and Yang, 2018
*W. confusa*	KR780676	Idli butter	India	Survival under hGIt conditions, binding properties, cholesterol removal, heat resistance and β-galactosidase activity, biofilm formation, antioxidant activities, inhibition of pathogenic biofilm formation	*In vitro* hemolytic activity, antibiotic resistance and DNase and gelatinase activity	Sharma et al., 2018
*W. cibaria*	Strains 13 and 16 Strains 28 and 29	Dosa batter Human infant feces	India	Survival under hGIt conditions, *in vitro* adhesion assay, adhesion to hydrocarbons, antimicrobial activity, in vitro cholesterol reduction, attenuation of lipopolysaccharide-induced pro-inflammatory stress in murine macrophages(RAW 264.7) and in human intestinal epithelial cells (Caco-2)	-	Singh et al., 2018
*W. cibaria*	FB069	Fermented salted shrimp	Korea	Prevention of proliferation in human colon cancer cells (Caco-2 and HCT116 cell lines) by symbiotic fermented soymilk with this strain and xylooligosaccharides	-	Le et al., 2020
*W. cibaria*	D29 and D30	Kimchi	Korea	Survival under hGIt conditions, antimicrobial spectrum, *in vitro* adhesion capacity, antioxidant activity, exopolysaccharide production	*In vitro* hemolytic activity, antibiotic resistance	Yu et al., 2019
*W. confusa*	WH2, Wh4, WH6, WH7	Horse feces	China	Acid tolerance, heat resistance, α-amylase inhibition test, antioxidant capacity, antimicrobial spectrum	Antibiotic resistance	Xia et al., 2019
*W. confusa*	F213	Human	Indonesia	Enhancement of intestinal epithelial barrier function in Caco-2 cell monolayers exposed to hydrogen peroxide to induce inflammatory bowel disease (exposed to both *W. confusa* F213 and *Lactobacillus rhamnosus* FBB81)	-	Fatmawati et al., 2020
*W. paramesenteroides*	FX1, FX2, FX5, FX9 and FX12	Fruits	India	Viability in low pH and sodium taurocholate, salt aggregation and autoaggregation, biofilm formation, in vitro adhesion to mucin	Antibiotic resistance	Pabari et al., 2020
*W. confusa*	YM5Y, YM5S1 and YM5S2	Healthy human feces	China	Survival under hGIt conditions, antimicrobial spectrum	*In vitro* hemolytic activity, antibiotic resistance	Wang et al., 2020b
*W. oryzae*	NABRII48, NABRII60, NABRII62, NABRII63, NABRII47	Rainbow trout	Spain	Survival under hGIt conditions, antimicrobial spectrum	*In vitro* hemolytic activity, antibiotic resistance, PCR detection of virulence factors	Mortezaei et al., 2020
*W. confusa* and *W. cibaria*	*W. confusa* 12, 16, 19, 111, 116, 117. *W. cibaria* 118, 123, 126, 132	Camel milk	Morocco	Survival under hGIt conditions, antimicrobial spectrum, cell surface hydrophobicity and autoaggregation, antioxidant activity, exopolysaccharide production. Technological characterization (acidifying capacity, proteolytic and lipolytic activities, diacetyl production, autolytic activity and heat resistance)	Antibiotic resistance, DNase and hemolytic activity	Mercha et al., 2020
*W. confusa* and *W. cibaria*	MD1 and MD2	Fermented batter	India	Survival under hGIt conditions, antimicrobial spectrum, cell surface hydrophobicity and autoaggregation, cholesterol removal, antioxidant activity, biofilm analysis by atomic force microscopy.	*In vitro* hemolytic activity, antibiotic resistance	Lakra et al., 2020a
*W. cibaria*	JW15	kimchi	Korea	Immune-modulating effects on murine macrophage cell line RAW 264.7	-	Park et al., 2019
*W. confusa*	MDM8	Wheat sourdough	Iran	Enhancement of γ-aminobutyric acid content in milk fermented with MDM8 alone or in co-culture with and Enterococcus faecium strain	Antibiotic resistance	Khanlari et al., 2021
*W. viridescens*	UCO-SMC3	Adult snails	Not specified	Glass adherence, adherence assay on HaCat cell line, resistance to gastric conditions, hydrogen peroxide production, lactic acid and bacteriocin detection, microbicidal activity on *Cutibacterium acnes* and *Staphylococcus aureus*, antagonistic activity on the adhesion of C. acnes and *S. aureus* in HaCat cells	Antibiotic susceptibility, hemolysis and gelatinase activities, cytotoxicity on HaCat cells	Espinoza-Monje et al., 2021
*W. confusa*	GCC_19R1	Fermented sour rice	India	Survival under hGIt conditions, antimicrobial spectrum, cell surface hydrophobicity and autoaggregation,	*In vitro* hemolytic activity, antibiotic resistance	Nath et al., 2021
*W. cibaria*	SP7 and SP19	Dairy cows	Kuwait	Survival under hGIt conditions, antimicrobial spectrum, and auto- and co-aggregation, carbohydrate fermentation patterns and exopolysaccharide production	*In vitro* hemolytic activity, antibiotic resistance	Patrone et al., 2021
*W. paramesenteroides*	MN2C2	Buffalo colostrum	Egypt	Anticancer and antioxidant activities of L-asparaginase produced by the strain on breast cancer, colorectal adenocarcinoma, hepatocellular carcinoma and lung cancer cell lines.	-	Amer et al., 2022
*W. cibaria*	CMU CMS1	saliva of healthy Korean children	Korea	Inhibition of the formation of multispecies colony biofilms (saliva-coated titanium disks)	-	Kang and Park, 2022
*W. cibaria*	KY10	Digestive tract of healthy shrimp	Thailand	Antimicrobial activity against *Vibrio parahaemolyticus*, survival under hGIt conditions, hydrophobicity,	Hemolytic activity, antibiotic susceptibility, biosafety evaluation in vivo (in shrimp)	Kanjan et al., 2022
*W. paramesenteroides*	MYOS5.1	Dairy products	India	Survival under hGIt conditions, antimicrobial spectrum, auto- and co-aggregation, biofilm formation, exopolysaccharide production and extraction, in silico prediction of antitumor activity	Antibiotic resistance, DNase activity	Yadav and Sunita, 2022
*W. hellenica*	D1501	Not specified	China	Neuroprotective effects on the hydrogen peroxide-stimulated oxidative damage model in a neural-like cell (PC12) by soybean whey fermented with the probiotic strain	-	Yin et al., 2022
*W. cibaria*	CMU	Saliva of healthy Korean children	Korea	Preventive effect and mechanism of action of CMU against *Streptococcus mutans* biofilm formation and periodontal pathogens (*Porphyromonas gingivalis, Fusobacterium nucleatum*, or *Prevotella intermedia*)	-	Kang et al., 2023a
*W. cibaria*	D29, D30, D31, B22	Kimchi	Korea	Antibacterial and antibiofilm effects on *Streptococcus mutans*, which causes dental caries	-	Kang et al., 2023b
*W. diestrammenae*	DSM 27940^T^	Gut camel cricket	Sud Korea	Antimicrobial spectrum, Survival under hGIt conditions, in vitro adhesion assay, auto-aggregation capacity, cell surface hydrophobicity, search of probiotic genes in the genome	Hemolytic activity, antibiotic susceptibility, search of virulence and antibiotic resistance genes in the genomes	Fanelli et al., 2023b
*W. uvarum*	B18NM42^T^	Grapes	Greece			
*P. beninensis*	LMG25373^T^	Fermented cassava	Africa			
*P. fabalis*	LMG 26217^T^	Cocoa bean fermentation	Brazil			
*P. ghanenis*	DSM 19935^T^	Cocoa bean fermentation	Ghana			
*P. fabaria*	LMG 24289^T^	Cocoa bean fermentation	Ghana			

^*T*^Type strain.

**Table 6:** Probiotic potential of *Weissella* strains by *in vivo* studies.

**Species**	**Strain**	**Source**	**Nation**	**Probiotic properties**	**References**
*W. cibaria*	Not specified	Indian fermented food	Korea	Capacity of lipoteichoic acid isolated from the probiotic strain in increasing cytokine production in human monocyte-like THP-1 cells and mouse splenocytes	Hong et al., 2016
*W. cibaria*	WIKIM28	Gatkimchi (kimchi made from mustard leaves)	Korea	Amelioration of atopic dermatitis-like skin lesions in BALB/c mice	Lim et al., 2017
*W. cibaria*	WD2	Fermented cassava and wara	Nigeria	Protective effect against cadmium and lead toxicities in rats	Ojekunle et al., 2017
*W. cibaria*	JW15	kimchi	Korea	Immunomodulatory potential in aged C57BL/6J mice	Park et al., 2017
*W. cibaria*	LW1, LW2 and LW3	Not specified	China	Inhibition of colonization and infection of *Staphylococcus aureus* in mammary glands of BALB/c mice	Wang L. et al., 2017
*W. cibaria*	JW15	kimchi	Korea	Enhancement of immune functions by increasing natural killer cell activity in non-diabetic humans (randomized, double-blinded, placebo-controlled study)	Lee et al., 2018
*W. cibaria*	JW15	kimchi	Korea	Immune enhancing effects on BALB/c mice immunosuppressed by cyclophosphamide	Park and Lee, 2018
*W. paramesenteroides*	WpK4	Nasal mucosa of piglets	Brazil	Reduction of the parasitic loads of gerbils infected with *Giardia lamblia*	Fonseca et al., 2019
*W. cibaria*	JW15	kimchi	Korea	Improved performance characteristics (decrease in the serum concentration of triglycerides and feces ammonia emissions; improvement of high-density lipoprotein cholesterol in serum and feces) in adult Beagle dogs	Sun et al., 2019
*W. cibaria*	CMU	saliva of healthy Korean children	Korea	Suppression of halitosis, colonization of the oral cavity, inhibition of the proliferation of oral bacteria causing malodor in beagles	Do et al., 2019
*W. cibaria*	CMU	saliva of healthy Korean children	Korea	Inhibition of enzymes producing volatile sulfur compounds and suppression of the mgl gene expression in the malodor causing oral bacterium *Porphyromonas gingivalis*	Kim et al., 2019
*W. cibaria*	CMU	saliva of healthy Korean children	Korea	Reduction of halitosis in humans (randomized, double-blind, placebo-controlled study)	Lee et al., 2020
*W. cibaria*	CMU	saliva of healthy Korean children	Korea	Reduction of periodontal tissue destruction in mice	Kim et al., 2020b
*W. cibaria*	CMU	saliva of healthy Korean children	Korea	Inhibitory effect on halitosis in human (randomized placebo-controlled study)	Kim et al., 2020a
*W. cibaria*	CMU	saliva of healthy Korean children	Korea	Improvement in the bleeding on probing and microbial environment in humans (randomized, double-blind, placebo-controlled study). Antimicrobial activity against *Fusobacterium nucleatum*	Kang et al., 2020
*W. confusa*	DD_A7	Not specified	Korea	Amelioration of inflammation response against Escherichia coli O157:H7 in zebrafish larvae	Dey and Kang, 2020
*W. paramesenteroides*	WpK4	Nasal mucosa of piglets	Brazil	Immunobiotic role in gut-brain axis by reduction of gut permeability, anxiety-like and depressive-like behaviors in murine models of colitis and chronic stress	Sandes et al., 2020
*W. paramesenteroides*	WpK4	Nasal mucosa of piglets	Brazil	Amelioration of the experimental amoebic colitis in BALB/c mice	Prado et al., 2020
*W. cibaria*	MW01	Chinese sauerkraut	China	Attenuation of the liposaccharide-induced dysfunction of intestinal epithelial barrier in a caco-2 cell monolayer model	Huang et al., 2020
*W. confusa*	JMC 1093	Nigerian fermented food condiment	Nigeria	Alleviation of formalin induced inflammation in rats by oral administration of the probiotic *W. confusa* strain and a *Pediococcus pentosaceus* strain	Oladajo and Oluwasola, 2021
*W. cibaria*	CMU	saliva of healthy Korean children	Korea	Elimination of the risk of developing dental caries from acid production in the oral flora in human (randomized, double-blind, placebo-controlled trial)	Kang et al., 2021
*W. cibaria*	MG5285	Not specified	Korea	Attenuation of fat accumulation in adipose and hepatic steatosis in high –fat diet-induced C57BL/6J obese mice	Choi et al., 2021
*W. confusa*	31-1	Not specified	Iran	Improvement of the growth performance, serum immune parameters, immune-related gene expression and intestinal microbiota in rainbow trout (*Oncorhynchus mykiss*)	Kahyani et al., 2021
*W. viridescens*	UCO-SMC3	Adult snails	Not specified	Protection against *Cutibacterium acnes* in Balb/c mice	Espinoza-Monje et al., 2021
*W. cibaria*	CMU CMS1	saliva of healthy Korean children	Korea	Therapeutic efficacy on allergic inflammation exacerbated by diesel exhaust particulate matter in a murine asthma model	Do et al., 2022
*W. cibaria*	II-1-59	Nigerian fermented food condiment	Nigeria	Immunomodulation and treatment of inflammation-induced anemia in Wistar rats	Oladejo and Oluwasola, 2022
*W. confusa*	VP30	Children's feces	Korea	Improvement of the functional laxative effects of milk fermented with the probiotic strain in loperamide-induced constipation in rats	Park et al., 2022b
*W. confusa*	CGMCC 19,308	Human feces	China	Antioxidant activity and prolongment of lifespan and enhancement of host defense against *S*. Typhimurium of the nematode *Caenorhabditis elegans*	Wang Q. et al., 2022
*W. cibaria*	C-10	Healthy crucian carp	China	Enhancement of the immunity against *Aeromonas veronii* infection in crucian carp by dietary supplementation with the *W. cibaria* strain and a *B. amyloliquefaciens* strain	Zhu et al., 2022
*W. cibaria*	CMU	saliva of healthy Korean children	Korea	Improvement of halitosis in human (randomized, double-blinded, placebo-controlled study)	Han H. S. et al., 2022
*W. confusa*	VP30	Children's feces	Korea	Amelioration of functional constipation in humans (randomized, double-blind, controlled human study)	Jin et al., 2023

Jang et al. (2021) performed a safety assessment of *W. cibaria* JW15 by phenotypic (antibiotic susceptibility, production of toxic metabolites, and hemolytic activity) and genotypic analyses (whole-genome sequencing and search of virulence and antibiotic resistance genes), confirming the safety of this strain. The probiotic potential and the safety of four Periweissella and two Weissella type strains were assessed by Fanelli et al. (2023b) using phenotypic and genotypic methods. The potential probiotic *P. beninensis*-type strain results the only safe candidate. Furthermore, these authors confirmed the necessity of assessing the probiotic potential and safety of weissellas and periweissellas on a strain-specific basis (Fanelli et al., 2023b). Among *Lactobacillaceae, Weissella* species are the second only to *Lacticaseibacillus rhamnosus* causing opportunistic (hospital-acquired) infections, but even in the case of *Lc. rhamnosus*, EFSA has expressed its opinion on the safety of certain strains of this species to be used as technological additive for all animal species (EFSA Panel on Additives Products or Substances used in Animal Feed et al., 2017, 2021).

## An update of the probiotic potential of *Weissella* and *Periweissella*

While until 2014 few studies investigated the probiotic potential of weissellas by way of *in vitro* and *in viv*o studies (Fusco et al., 2015), from 2014 up to date, the number of such studies has increased greatly (Tables 5, 6), but, as reported in Table 5, those *in vivo* studies mainly involved *W. cibaria* strains, apart from some *W. confusa* and *W. paramesenteroides* strains and one *W. viridescens* strain. However, in addition to the study of Park et al. (2022a), who demonstrated the functional laxative effects of milk fermented with a probiotic *W. confusa* strain in loperamide-induced constipation in rats, none of the *in vivo* studies administered food or feed containing weissellas strains to animals or humans.

### Antimicrobial activity of weissellas

According to Fusco et al. (2015), up to the year 2014, 12 articles were published on the screening of *Weissella* strains for their antimicrobial activity. From 2014 up to date, further investigations have investigated weissellas for their antagonistic activity against pathogens (Jang et al., 2016; Shah et al., 2016; Das Purkhayastha et al., 2017; Ye K. et al., 2018; Yu et al., 2018; Dey et al., 2019; Tenea and Israel Lara, 2019; Dinoto et al., 2021; Yeu et al., 2021; Styková et al., 2022; Yang C. et al., 2022; Yao D. et al., 2022; El-Mekkawy et al., 2023; Fanelli et al., 2023b; Table 5). In addition, Kang and Park (2022) demonstrated the *in vitro* inactivation of respiratory viruses, namely, human respiratory syncytial virus (RSC) and the influenza A virus (H1N1) and rotavirus by the oral probiotic strain *W. cibaria* CMS1, isolated from the saliva of Korean children with healthy oral cavity.

As reported by Fusco et al. (2015), up to 2014, six bacteriocins were detected in five *Weissella* strains. From 2014 up to date, further bacteriocins have been discovered in these bacteria. In particular, weissellicin 110 produced by a strain isolated from the yan-dong-gua (fermented wax gourd), namely, *W. cibaria* 860106, was characterized by Wu et al. (2015). The bacteriocin A3 produced by *W. confusa* A3, a strain of dairy origin, was isolated and characterized by Goh and Philip (2015). This bacteriocin inhibited the growth of strains of *P. aeruginosa, Bacillus cereus, Enterococcus faecium, Micrococcus luteus, E. coli*, and *Lactococcus lactis* (Goh and Philip, 2015). Two putatively novel bacteriocins, namely, bacteriocin 7193A and bacteriocin 7293B, produced by *W. hellenica* BCC 7293, isolated from Nham (Thai fermented pork sausage) were isolated by Woraprayote et al. (2015). Apart from some Gram-positive strains including *Staphylococcus aureus* ATCC 23235 and *S. aureus* ATCC 25923, both bacteriocins were found active against Gram-negative foodborne pathogens such as *Salmonella enterica* serovar Typhimurium, *Aeromonas hydrophila, Escherichia coli*, and *Pseudomonas aeruginosa*.

*W. confusa* MBF8-1, isolated from a Indonesian home-made soya product, revealed bacteriocin-like inhibitory substance (BLIS) activity against some Gram-positive bacteria including six *W. confusa* strains, two *W. cibaria* strains, a *Leuconostoc mesenteroides* strain, a *Macrococcus luteus*, and a *Lactococcus lactis* strain (Malik et al., 2016). Malik et al. (2016) demonstrated that the so called weissellicin MBF was encoded by a large plasmid, pWcMBF8-1. Subsequently, Sartono et al. (2019) demonstrated spermicidal and antibacterial activity against the indicator bacterium *Leuconostoc mesenteroides* of the bacteriocin-like peptides of *W. confusa* MBF8-1. BLIS that is active against non-specified Gram-positive and Gram-negative bacteria was found also in *W. confusa* LM85 by Kaur and Tiwari (2016).

Dubey and Jeevaratnam (2018) demonstrated the antimicrobial activity of *W. confusa* AJ79, isolated from fermented butter, against numerous pathogenic bacteria such as *Citrobacter freundii, Aeromonas hydrophila, Bacillus cereus, B. subtilis, Mycobacterium smegmatis, E. coli, B. licheniformis, L. monocytogenes, Clostridium sporogenes, Klebsiella pneumoniae, C. perfringens, Micrococcus luteus, Pseudomonas aeruginosa, Vibrio parahaemolyticus, S. aureus, S. epidermidis*, and *Proteus vulgaris*. These authors also isolated and characterized the relevant class II bacteriocin named BAC79 from this strain (Dubey and Jeevaratnam, 2018). Kariyawasam et al. (2019) successfully investigated the antilisterial effect of *W. cibaria* D30, isolated from Korean kimchi, when used as protective culture in the production of cottage cheese, but they did not ascertain to what kind of antimicrobial compound this effect was due.

Teixeira et al. (2022) sequenced the genome of *W. cibaria* W25, isolated from a Brazilian pasture samples of a Brazilian dairy farm, and found that this strain had the possibility of producing two different bacteriocins. Thereafter, the same authors (Teixeira et al., 2023) demonstrated a putative bacteriocinogenic activity of *W. cibaria* W25 against *Salmonella enterica* Newport, *Kocuria rhizophila, Listeria innocua*, and *E. coli* strains. The same antimicrobial spectrum was observed also in the neutralized supernatant of *W. cibaria* W42, isolated from the soil of a Brazilian dairy farm (Teixeira et al., 2023). Moreover, Teixeira et al. (2023) sequenced also the genomes of *W. cibaria* W42 and *W. cibaria* W21 isolated from pasture samples of a Brazilian dairy farm. In these strains, they also found the putative gene for a bacteriocin identified as bacteriocin_IIc (Teixeira et al., 2023), but the strain W21 lacks the transport-related gene and its neutralized supernatant lost antimicrobial activity, whereas the supernatant of W25 and W42 lost the antimicrobial activity only when treated with proteinase K (Teixeira et al., 2023).

### Heavy metal biosorption by weissellas

Apart from antimicrobial activity of weissellas, the study of Li et al. (2021) also demonstrated the cadmium biosorption of *W. viridescens* ZY-6, isolated from fermented pickles, whereas Kinoshita et al. (2016) demonstrated the biosorption properties of *W. viridescens* MY 205 isolated from bovine intestine for the periodic group 12 metals cadmium (Cd), mercury (Hg), and zinc (Zn). Considering the detrimental effects of heavy metal pollution of food and beverages on human and animal health, the role of weissellas as heavy metal sorbents may be of high importance.

### Aflatoxin-binding activity of weissellas

Weissellas may also play an important role in binding of aflatoxins. Kavitake et al. (2020), for example, demonstrated that a strain of *W. confusa*, isolated from an Indian traditional fermented food (Idli batter), produced a galactan exopolysaccharide with aflatoxin B1-binding activity. As in the case of heavy metals discussed above, this may prevent absorption of the aflatoxin in the human body once the food with the bacteria is ingested. However, more research in this would be required.

### Exopolysaccharides produced by weissellas

Among the metabolites produced by weissellas, exopolysaccharides (EPSs) play an important role in several beneficial and technological functions. Since 2015, when Fusco et al. (2015) reviewed the research published on weissellas' EPS up to that date, a plethora of papers were published dealing with the characterization of weissellas producing EPS (Table 7). Mainly homo- but also heteropolysaccharides have been found in *W. cibaria* and *W. confusa* strains of various origin, with technological and functional properties (Table 7). The latter mainly include antioxidant, antibacterial, antifungal, anti-inflammatory, and prebiotic functions, which have been demonstrated *in vitro* (Table 7), while few *in vivo* studies have demonstrated the amelioration of functional constipation in rats and humans (Table 7).

**Table 7:** Exopolysaccharides (EPS) produced by *Weissella* strains documented from 2015 up to date.

**Species**	**Strain**	**Origin**	**Type of EPS**	**Properties**	**References**
*W. cibaria*	RBA12	Pummelo (*Citrus maxima*)	Glucansucrase, glucan and oligosaccharides	-	Baruah and Goyal, 2015
*W. confusa*	AJ53	Fermented Uttapam batter supplemented with Piper betle L. leaves	Unbranched linear (α-1 → 6 linked) dextran	Prevention of syneresis, hydration properties, emulsification activity, and flocculation power, cryoprotectant activity, antioxidant activity	Dubey and Jeevaratnam, 2015
*Weissella* spp.	-	African spontaneously fermented Malian sour milk or cassava products from Ivory Coast	Dextran, glucan, levan and fructan type polymers	Water solubility	Malang et al., 2015
*W. confusa*	MBF8-1, MBF8-2, MBFCNC	Indonesian beverages	Fructan	Viscosity	Malik et al., 2015
*W. cibaria*	KJ742706	Fermented Sauropus androgynus	Linear dextran with α-(1 → 6) glycosidic bonds	Rheology, syneresis, thermal stability (280°C), cytotoxicity	Vasanthakumari et al., 2015
*W. cibaria*	RBA12	Pummelo (Citrus maxima)	Dextran composed of 3% α - (1 → 3) and 97% α - (1 → 6) linkages	Prebiotic activity	Baruah et al., 2017
*W. confusa*	EPSWWC (wild type)	-	Heteropolysaccharide containing galactose as main sugar	Immunomodulatory activity and antioxidant properties	Adebayo-Tayo et al., 2018
*W. confusa*	OF126	Ogi (Nigerian fermented cereal pudding)	Homopolysaccharide containing glucose monomers with α - (1 → 3) branched linkage and α - (1 → 6) linkage	Antioxidant properties and water solubility	Adesulu-Dahunsi et al., 2018a
*W. cibaria*	GA44	Gari (Nigerian fermented cassava mash)	Heteropolysaccharide mainly composed of rhamnose and glucose units	Antioxidant properties	Adesulu-Dahunsi et al., 2018b
*W. confusa*	321	Yellow corn flour	Homopolysaccharide containing glucose monomers with α - (1 → 3) branched linkage and α - (1 → 6) linkage	Thermal stability (288°C)	Petrovici et al., 2018
*W. cibaria*	YB-1	Chinese pickled cabbage	Dextran with α - (1 → 6) linkages and few α - (1 → 3) linked branches	Water-holding capacity, water solubility, antioxidant properties and emulsifying activity	Ye G. et al., 2018
*W. confusa*	QS813	Chines traditional sourdough	Low branched dextran with 97% of α - (1 → 6) linkages	Rheology, syneresis	Tang et al., 2018, 2019
*W. cibaria*	SJ14	*Sichuan paocai*	Heteropolysaccharide rich in mannose and containing mannose, arabinose, galactose, glucose, rhamnose and xylose	Antioxidant properties	Zhu et al., 2018
*W. confusa*	W4	Kila (traditional Algerian cheese)	α - (1 → 6) glycosidic	Antioxidant properties, rheology, syneresis	Benhouna et al., 2019
*W. confusa*	KR780676	Idli batter	Linear galactan homopolysaccharide containing α - (1 → 6) galactose units	Thermal stability, water solubility, oil-holding capacity, flocculation properties, emulsifying activity, aflatoxin binding ability, prebiotic properties	Kavitake et al., 2019, 2020; Devi et al., 2021
*W. confusa*	VP30	Young children feces	Dextran 96.5% α - (1 → 6) glycosidic bonds and 3.5% α - (1 → 3) branches	*-* *In vivo* biofunctionality for the alleviation of loperamide-induced constipation in rats *In vivo* amelioration of functional constipation in humans (randomized, double-blind, controlled study)	Jin et al., 2019, 2023; Park et al., 2022a
*W. confusa*	MD1	Dosa batter	Mannan with α-(1,6)-linked mannose units	Antibiofilm activity, antioxidant properties	Lakra et al., 2020b
*W. cibaria*	KRK005	Young radish	Glucooligosaccharides	Prebiotic and immunostimulatory activities	Kwon and Park, 2021
*W. cibaria*	MD2	Fermented batter	Fructan with β- (1 → 2)linkages	Thermal stability (279.9°C), *in vitro* and *in vivo* antioxidant properties, enhancement of *Caenorhabditis elegans*	Lakra et al., 2021
*W. confusa*	–	Well-heated meat goulash	Dextran	Hemolytic activity	Siavoshi et al., 2021
*W. confusa*	XG-3	Not specified	Linear dextran composed of α - (1 → 6)-linked D-pyranose residues	Thickener and stabilizing activities, antioxidant activity, thermal stability (306.8°C), prebiotic activity	Zhao D. et al., 2021
*W. cibaria*	NC516.11	Distiller grains of Chinese Baijiu	Glucan with 93.46% α - (1 → 6) D-glucose linkages and 6.54% α - (1 → 3) D-glucose linked branches	Water-holding capacity and rheological properties	Li et al., 2022
*W. cibaria*	FMy 2-21-1	Not specified	Linear α-1,6 dextran	Corrosive coating inhibition	Lobo et al., 2022
*W. cibaria*	SY003	kimchi	Heteropolysaccharide composed of glucose (38.95%) and galactose (61.04%) linked through α-D-Glc-(1 → and → 6)-β-D-Gal-(1 → linkage)	Antioxidant activity, non-toxicity, and biocompatibility	Park et al., 2022b
*W. confusa*	W-16	Pre-fermentation liquid of wheat	Dextran with (1 → 3)-linked α-D- glucose units as braches at levels of 9.2%	Thermal stability (300°C) Use in combination with whey protein isolate as fat substituting in low-fat mayonnaise samples	Yalmanci et al., 2022, 2023
*W. cibaria*	-	Vegetable juice	Linear glucan containing α-1,6 glycosidic bond	Prebiotic properties	Wang et al., 2023

## Conclusion and outlook

In the last decades the number of articles published on weissellas is increased enormously. Novel species have been discovered, the taxonomy of the genus has changed so that a new genus, namely *Periweissella*, has been derived from the previous genus *Weissella*, and new insights into the safety, biotechnological, and probiotic potential of weissellas and periweissellas have been provided. Regarding their technological characteristics, the weissellas and periweissellas have excellent potential with reference to their proteolytic and saccharolytic, as well as antimicrobial and EPS production activities, thus contributing greatly to the safety and functionality of the products. As for the safety, biotechnological, and probiotic potential, most studies focused on weissellas, mainly *W. confusa* and *W. cibaria*, but the most recent studies are focusing also on periweissellas and other species of the *Weissella* genus. Apart from one safety study carried out on 46 *W. confusa* strain allowing to define this species as safe, for the other *Weissella* and *Periweissella* species the safety assessment was carried out on few strains some of which resulted to be unsafe. These groups of LAB have a high potential for biotechnological application, and the probiotic potential of numerous strains is being demonstrated. However, for their application in food, a strain-based assessment of their safety still remains mandatory.

## Author contributions

VF: Conceptualization, Methodology, Project administration, Writing – original draft, Writing – review & editing. DC: Writing – original draft, Writing – review & editing. FF: Writing – original draft, Writing – review & editing. MM: Writing – original draft, Writing – review & editing. CR: Writing – original draft, Writing – review & editing. CF: Funding acquisition, Writing – original draft, Writing – review & editing.
